# Inhalation of lung spheroid cell secretome and exosomes promotes lung repair in pulmonary fibrosis

**DOI:** 10.1038/s41467-020-14344-7

**Published:** 2020-02-28

**Authors:** Phuong-Uyen C. Dinh, Dipti Paudel, Hayden Brochu, Kristen D. Popowski, M. Cyndell Gracieux, Jhon Cores, Ke Huang, M. Taylor Hensley, Erin Harrell, Adam C. Vandergriff, Arianna K. George, Raina T. Barrio, Shiqi Hu, Tyler A. Allen, Kevin Blackburn, Thomas G. Caranasos, Xinxia Peng, Lauren V. Schnabel, Kenneth B. Adler, Leonard J. Lobo, Michael B. Goshe, Ke Cheng

**Affiliations:** 1 Department of Molecular Biomedical Sciences, North Carolina State University, Raleigh, NC 27695 USA; 2 Comparative Medicine Institute, North Carolina State University, Raleigh, NC 27695 USA; 3 Department of Molecular and Structural Biochemistry, North Carolina State University, Raleigh, NC 27695 USA; 4 Joint Department of Biomedical Engineering, University of North Carolina at Chapel Hill and North Carolina State University, Raleigh, NC 27695 USA; 5 Department of Biological Sciences, North Carolina State University, Raleigh, NC 27695 USA; 6 Division of Cardiothoracic Surgery, University of North Carolina at Chapel Hill, Chapel Hill, NC 27599 USA; 7 Bioinformatics Research Center, North Carolina State University, Raleigh, NC 27695 USA; 8 Department of Clinical Sciences, North Carolina State University, Raleigh, NC 27695 USA; 9 Division of Pulmonary Diseases and Critical Care Medicine, University of North Carolina at Chapel Hill, Chapel Hill, NC 27599 USA

**Keywords:** Stem cells, Respiratory tract diseases, Translational research

## Abstract

Idiopathic pulmonary fibrosis (IPF) is a fatal and incurable form of interstitial lung disease in which persistent injury results in scar tissue formation. As fibrosis thickens, the lung tissue loses the ability to facilitate gas exchange and provide cells with needed oxygen. Currently, IPF has few treatment options and no effective therapies, aside from lung transplant. Here we present a series of studies utilizing lung spheroid cell-secretome (LSC-Sec) and exosomes (LSC-Exo) by inhalation to treat different models of lung injury and fibrosis. Analysis reveals that LSC-Sec and LSC-Exo treatments could attenuate and resolve bleomycin- and silica-induced fibrosis by reestablishing normal alveolar structure and decreasing both collagen accumulation and myofibroblast proliferation. Additionally, LSC-Sec and LSC-Exo exhibit superior therapeutic benefits than their counterparts derived from mesenchymal stem cells in some measures. We showed that an inhalation treatment of secretome and exosome exhibited therapeutic potential for lung regeneration in two experimental models of pulmonary fibrosis.

## Abstract (web summary)

Idiopathic pulmonary fibrosis (IPF) is a fatal lung disease and adult lung spheroid cells have been shown to promote regeneration in animal models of IPF. Here the authors show that the secretome and exosomes of lung spheroid cells is effective as inhalation treatment in rodent models of lung injury and fibrosis and superior to the counterparts derived from mesenchymal stem cells.

## Introduction

Our entire body and all its organs are covered with a protective layer of epithelial cells. The epithelial cells in our lungs not only facilitate oxygen exchange but also defend against continuous exposure to inhaled irritants and toxins. Fortunately, our lungs have the ability to undergo facultative regeneration because of the resident stem and progenitor cell populations that are essential for maintaining tissue homeostasis and repair in response to acute and/or chronic lung injuries. Our previous studies have shown that therapeutic adult lung cells, termed LSCs, contain a heterogeneous population of cells expressing lung epithelial (Epcam, AQP5, ProSPC, and CCSP) and mesenchymal (CD90 and CD105) markers^1–3^. The purpose of the present study is to examine the therapeutic effects of lung spheroid cell secretome (LSC-Sec) in pulmonary fibrosis. It has been reported that the mesenchymal stem cell secretome (MSC-Sec), or conditioned media, indeed reproduces the therapeutic effects seen in MSC cell therapy in some diseases, such as osteoarthritis, bronchopulmonary dysplasia, and multiple sclerosis^4–6^.

Respiratory-related morbidity and mortality are on the rise^7^. While some chronic lung injuries have been linked to environmental factors or lifestyle choices such as smoking, the cause of Idiopathic Pulmonary Fibrosis (IPF) remains unknown (as the name implies). IPF is actively being studied by many groups as it is a progressive and fatal form of interstitial lung disease, characterized by fibroblastic foci, alveolar honeycombing, and persistent, unremitting fibrosis that ultimately leads to the destruction of lung architecture and fulminant respiratory failure^8,9^. The exact pathological mechanism(s) and cause(s) of IPF are unknown, resulting in poor prognosis. There remain few treatment options for IPF. To date, only two Food and Drug Administration (FDA) approved therapies exist: pirfenidone and nintedanib. These drugs, however, are palliative and merely delay disease progression^10,11^. They do not halt or reverse the fibrosis that is already established.

As new treatment strategies continue to evolve, cell-based therapies have emerged as promising options for a number of diseases^12,13^. Although stem cells have beneficial effects, their clinical applications face many challenges, including extensive labor, high cost, and safety concerns. There are concerns over cell stability, as stem and progenitor cells are susceptible to transformations during prolonged in-vitro cell cultures. The possibility for cell instability also raises tumorigenic and immunogenic risks. In addition, cell therapy products need to be carefully preserved and processed before clinical applications. One viable way of mitigating these safety concerns while retaining the therapeutic benefits of the cells would be to use secretome or conditioned media in place of the actual cells. Cumulating evidence indicates that the regenerative ability of adult stem cells is primarily due to their paracrine activity, which is administered through these secretions^1,14,15^. Therefore, our study will include MSC-Sec as a comparator treatment. Our over-arching hypothesis is that lung spheroid cell secretome (LSC-Sec) promotes lung repair in pulmonary fibrosis, in a fashion superior to its MSC counterpart.

## Results

### Cell-secretome mitigates bleomycin-induced fibrosis and apoptosis

**Fig. 1:**
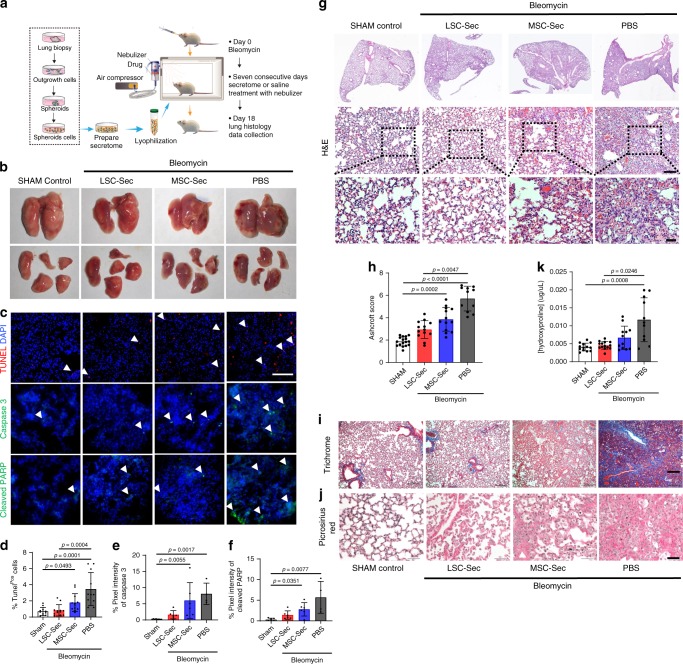
LSC-Sec inhalation reverses alveolar epithelial cell damage caused by Bleomycin injury. **a** The schematic of LSC-Sec procedure and study design; *n* = 12 biological independent animals per group. **b** Macroscopic view of the explanted lungs at study endpoint. **c** Representative TUNEL (top), Caspase 3 (middle), and cleaved PARP (bottom) immunostaining of apoptotic cells for each treatment group. **d**–**f** Quantification of percent of Tunel (**d**) *n* = 12 biological independent animals, Caspase 3 (**e**) *n* = 6 biological independent animals, and cleaved PARP (**f**) positive cells, *n* = 6 biological independent animals; Scale bar = 100 μm; each dot represents data from one animal; *n* = 6–12. **g** Representative H&E staining. Top: Scale bar = 500 μm Bottom: Scale bar = 200 μm. **h** Quantification of fibrosis by Ashcroft score; each dot represents data from one animal; *n* = 12 biological independent animals. Ashcroft score was performed by averaging the score from two blinded and one non-blinded scorer. **i** Representative Gomori’s trichrome staining; muscle fibers (red), collagen (blue), nuclei (black-purple), and erythrocytes (red). Scale bar = 1000 μm. **j** Representative picrosirius red staining; Collagen types I and III (red). Scale bar = 50 μm. **k** Quantification of pulmonary hydroxyproline levels; each dot represents data from one animal; *n* = 12 biological independent animals. Throughout, data are mean ± s.d. *P*-value as indicated by non-parametric one-way ANOVA.

To test the impact of LSC-Sec on lung repair and fibrosis, we created a pulmonary fibrosis (PF) model using a single, high-dose, intratracheal (i.t.) bleomycin (Bleo) injection in immunocompetent CD1 mice. Body weight was monitored throughout the study as a measure of disease burden (Supplementary Fig. 1a). Our goal was to treat fibrosis, not inflammation; therefore, we allowed the initial inflammatory phase to pass and fibrosis to set in before starting treatment. Fibrosis progression in IPF patients is widely unknown and is vastly different from one patient to the next, but our previous experience with the Bleo model showed that inflammation peaks around day seven post injection and transitions to a more fibrogenic phase around day nine dominated by deposition of extracellular matrix and destruction of the alveolar structures^1,16^. Therefore, we waited until day ten after the inflammation phase has passed and fibrosis has started to begin intervention to ensure that we are in the therapeutic phase of the disease model (Fig. 1a). At day 10, the mice were given inhalation treatment using a nebulizer for seven consecutive days with a dose of 10 mg of secretome protein per kg of body weight, or an equal volume of PBS. To verify that nebulized substances were able to reach the distal lung, we tested mice with nebulized methylene blue dye. We immediately harvested lungs from one group of animals after one nebulization treatment for histological examination and verified that the dye indeed reached the distal lung (Supplementary Fig. 1b).

After the initial histological examination, all groups that received Bleo showed hemorrhagic necrosis (Fig. 1b), which decreased with either LSC-Sec or MSC-Sec treatment. Since Bleo induces DNA damage, we examined the effects of cell secretome treatment on apoptosis (Fig. 1c–f and Supplementary Fig. 2). LSC-Sec treatment led to a reduction in apoptosis in the lungs as compared to the PBS treated group (Fig. 1d–f) and bleomycin induced apoptosis occurs in a variety of lung epithelial and mesenchymal cell types (Supplementary Fig. 2). Both LSC-Sec and MSC-Sec treatments reduced fibrosis by preserving alveolar epithelial structures (Fig. 1g–h) and reduced collagen deposition (Fig. 1i–k). Hematoxylin and eosin (H&E) staining and corresponding Ashcroft score revealed that only LSC-Sec was able to reduce the fibrotic area and reverse the alveolar epithelial damage back to healthy (sham control) level.

### Secretome inhalation promotes vascular and alveolar repair

**Fig. 2:**
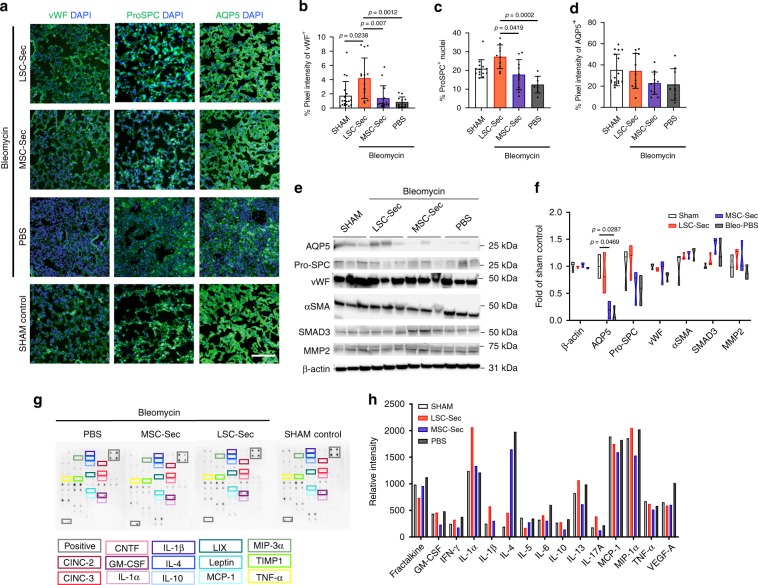
LSC-Sec inhalation treatment promotes alveolar repair. **a** Representative immunostaining of lung sections for von Willebrand Factor (vWF), pro-surfactant protein C (Pro-SPC) and aquaporin 5 (AQP5). Scale bar = 100 μm. **b**–**d** Quantification of percent pixel intensity of vWF+ (**b**), percent ProSPC+ nuclei (**c**), and percent pixel intensity of AQP5+ (**d**); each dot represents data from one animal; *n* = 12 biological independent animals. **e**–**f** Immunoblot analysis of aquaporin 5 (AQP5), pro-surfactant protein C (Pro-SPC), von Willebrand Factor (vWF), alpha smooth muscle actin (αSMA), SMAD3, matrix metalloproteinase 2 (MMP-2), and beta-actin loading control (B-actin) from lung protein lysate (**e**) with corresponding quantification of protein levels as fold of sham control (**f**); each dot represents data from one animal; *n* = 3 biological independent animals. **g**–**h** Representative cytokine array with quantification of relative intensity (**g**) and corresponding quantification of relative intensity (**h**). Throughout, data are mean ± s.d. *P*-value as indicated by non-parametric one-way ANOVA.

The alveoli are terminal structures of the distal airway. The alveolar epithelium is comprised of alveolar type 1 epithelial cells (AT1), which mediate gas exchange, and alveolar type 2 epithelial cells (AT2), which produce and release pulmonary surfactants, antioxidants, cytokines/chemokines, and other molecules important for the lung’s defense, response to insult, and homeostasis. Therefore, we examined the changes in AT1 and AT2 distribution in response to secretome treatment post-Bleo injury to assess epithelial damage and rescue (Fig. 2a). Immunostaining of AT1-marker aquaporin 5 (AQP5) showed that LSC-Sec treatment was able to reverse the epithelial damage caused by Bleo (Fig. 2a–d). However, immunoblots of lung protein lysate showed a significant increase of AQP5 proteins in LSC-Sec treated group compared to the PBS treated group (Fig. 2e, f). In addition, LSC-Sec treatment significantly increased the proliferation of surfactant protein C (ProSPC^+^)-positive AT2 cells (Fig. 2a–c), which proliferate and differentiate into AT1 cells. This response still occurred with MSC-Sec treatment, but to a lower extent which was not significantly different from the PBS group. Only LSC-Sec treatment was able to increase the expression of von Willebrand factor (vWF^+^)-positive vasculatures in the PF lungs (Fig. 2a, b). Immunoblots of ProSPC and vWF lung protein levels showed an increasing trend in the secretome treated groups compared to the PBS group (Fig. 2e, f).

In parallel, we examined the fibrotic response by measuring protein levels of alpha-smooth muscle actin (αSMA) (Fig. 2e, f), a myofibroblast marker indicative of IPF. LSC-secretome treated groups showed a decreasing trend in αSMA when compared to PBS treated controls. The protein expression of matrix metalloproteinase (MMP) 2 in lung tissue showed a trending increased in both secretome groups compared to the PBS group. We employed a cytokine array to access systemic cytokine expression as a measure of immunogenicity. Interestingly, LSC-Sec showed a trending decrease in pro-inflammatory IL-4 expression compared to the PBS controls (Fig. 2g, h). IL-4 is an important mediator of pro-inflammatory functions in respiratory conditions such as asthma^17–19^.

### LSC secretome therapy in silica-induced pulmonary fibrosis

**Fig. 3:**
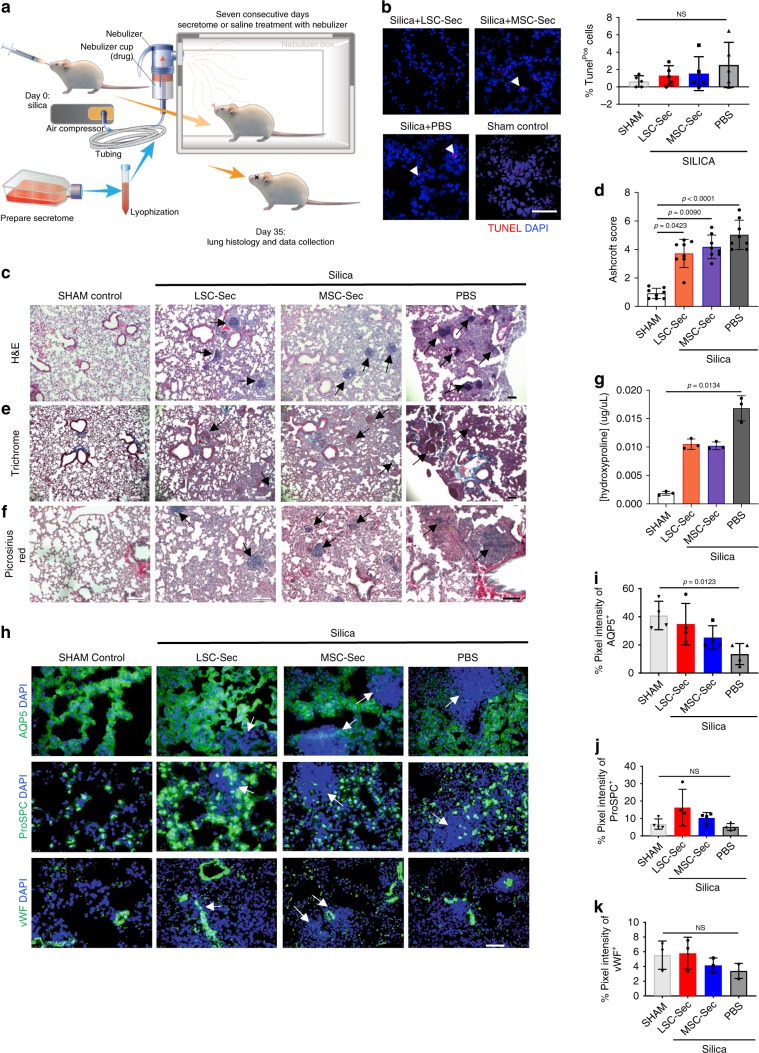
Lung repair and fibrosis in mice after silica-injury. **a** The experimental study schematic of the silica-induced fibrosis study in A/J mice; *n* = 10 biological independent animals per group. **b** Representative TUNEL staining of apoptotic cells for each treatment group and quantification of percent of Tunel positive cells; Scale bar = 50 μm; each dot represents data from one animal; *n* = 5 biological independent animals. **c** Representative H&E staining. Scale bar = 100 μm. **d** Quantification of fibrosis by Ashcroft score; each dot represents data from one animal; *n* = 8 biological independent animals. Ashcroft score was performed by averaging the score from two blinded and one non-blinded scorer. **e** Gomori’s trichrome staining: muscle fibers (red), collagen (blue), nuclei (black-purple) and erythrocytes (red). Scale bar = 100 μm. **f** Representative picrosirius red staining; Collagen types I and III (red). Scale bar = 100 μm. **g** Quantification of pulmonary hydroxyproline levels; each dot represents data from one animal; *n* = 4 biological independent animals. **h** Representative immunostaining of lung sections for aquaporin 5 (AQP5), pro-surfactant protein C (Pro-SPC) and von Willebrand Factor (vWF) Scale bar = 50 μm. **i**–**k** Quantification of percent pixel intensity of AQP5+ (**i**), ProSPC+ (**j**), and vWF+ (**k**). Each dot represents data from one animal; *n* = 4 biological independent animals. Throughout, data are mean ± s.d. *P*-value as indicated by non-parametric one-way ANOVA; NS, not significant.

To test whether the regenerative effects of LSC-Sec could be applied to other models of lung injury, we used the well-established silica model of induced pulmonary fibrosis^20^. Unlike Bleo, a biochemical agent that causes direct cellular injury, instillation of fine silica particles into the lungs causes fibrotic nodules to develop around the particles. Again, we treated the mice during the fibrotic phase, not the inflammatory period that precedes it. For the silica model, we waited until day 28 post-silica instillation to start treatment. Like the previous Bleo study, we administered inhalable cell secretome or saline for seven consecutive days using a nebulizer (Fig. 3a).

The physical presence of the particles embedded in the lungs aided in the localization and visualization of the fibrotic response. There was no significant difference in any of the treated groups regarding effects on apoptotic cells (Fig. 3b). However, LSC-Sec was able to significantly decrease the severity of the fibrosis as compared to the PBS treated group (Fig. 3c, d). The fibrotic tissues around the silica-induced nodules were less intense and widespread but persisted in both LSC-Sec and MSC-Sec treated groups (Fig. 3c–f). Cell secretome treatment also reduced collagen deposition and reduced alveolar epithelial damage, compared to the PBS treatment, although collagen deposition was still significantly higher than that of the healthy sham controls (Fig. 3f, g).

Examination of the AQP5^+^ AT1 cells and ProSPC^+^ AT2 cells revealed a non-significant decline in both alveolar markers in all silica-injured lungs (Fig. 3h–j). However, LSC-Sec was able to reduce the alveolar epithelial damage, compared to the PBS group, by promoting ProSPC^+^ AT2 cell expression and maintaining the AQP5^+^ AT1 cell population. As expected, the fibrotic nodules were absent of AQP5 expressing AT1 cells but still contained limited expression of ProSPC^+^ and vWF^+^ cells (Fig. 3h–k).

### Protein composition of LSC-secretome and exosomes

**Fig. 4:**
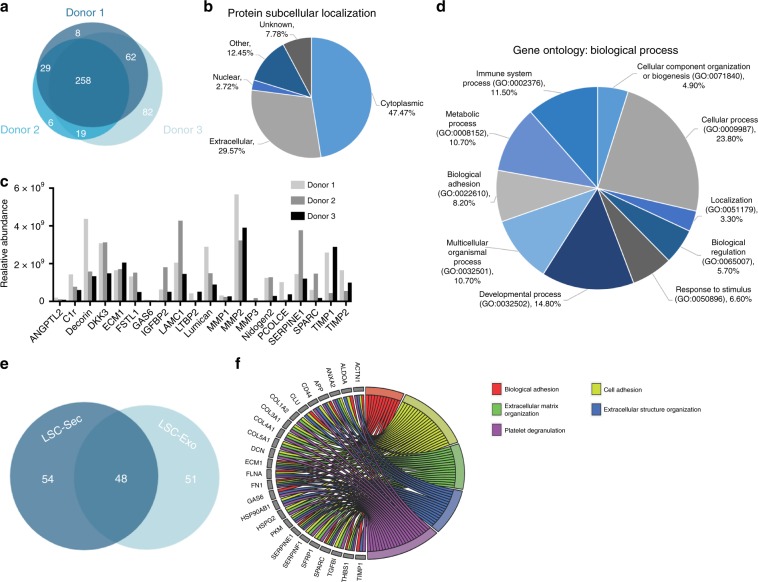
Proteomic analysis of LSC-Sec and LSC-Exo. **a** Venn diagram of proteins identified in LSC-Sec derived from three donors. **b** Pie chart of protein subcellular location of all common proteins in all three LSC-Sec. **c** Relative abundance of 20 of the 103 extracellular proteins identified in all three LSC-Sec. **d** Gene ontology pie chart of biological process associated with the shared extracellular proteins. **e** Venn diagram of extracellular proteins identified in LSC-Sec versus LSC-Exo. **f** Gene ontology chord chart of biology process associated with the shared LSC-Exo proteins.

To better understand the molecular processes that mediate the observed regenerative abilities of LSC-Sec, we sought to define its proteomic composition. We used pooled secretome from three different donor LSC lines (Supplementary Table 1) and employed liquid chromatography-tandem mass spectrometry (LC/MS/MS) analysis to examine their proteome and compare them to well-reported stem cell secretome. Interestingly, the three LSC lines had remarkably similar proteomes, considering that the cell lines were derived from individuals of different sex, race, and age (Fig. 4a). Of the shared proteins, 29.6% were annotated as extracellular proteins with known membrane receptors for secretion, and 47.5% were annotated as cytoplasmic proteins with no known secretion pathways (Fig. 4b).

The high percentage of cytoplasmic proteins prompted us to verify whether the proteins identified in LSC-Sec were truly proteins secreted from the cells or released from dead cells. Thus, we compared the identified cytoplasmic proteins from LSC-Sec with those proteins identified from lysed cells (Supplementary Fig. 3a–c). As expected, when LSCs were lysed, the majority of the proteome consisted of cytoplasmic proteins (65%) and only 5% of extracellular proteins. When comparing the cytoplasmic proteins detected in the LSC-Sec with the lysed cells, we found that only 56 out of 222 proteins (~25%) classified as cytoplasmic proteins in the secretome could be from potentially lysed or dead cells. These findings suggest that the other ~75% of the proteins annotated as cytoplasmic proteins identified in LSC-Sec are proteins that are released from the cells via a mechanism or pathway that is currently unknown.

The majority of the 102 common extracellular proteins identified in all three donors were growth factors and extracellular matrix-related proteins (Supplementary Data 1) and even though the LSC-Sec consisted of human proteins, many of which have high homology in mice and rats (Supplementary Data 2). Most notably from this list were proteins involved in Wnt signaling (dickkopf WNT signaling pathway inhibitor 3 [DKK3]), the complement system (complement C1r subcomponent [C1r] and decorin), angiogenesis (angiopoietin-like 2 [ANGPTL2] and extracellular matrix protein 1[ECM1]), lung development (follistatin-like 1 [FSTL1] and nidogen 2), cell proliferation (growth arrest-specific 6 [GAS6] and insulin like growth factor binding protein 2 [IGFBP2]), and extracellular matrix (ECM) remodeling (matrix metalloproteinase [MMP] 1, 2, 3, tissue inhibitor of metalloproteinase [TIMP] 1, 2, procollagen C-endopeptidase enhancer [PCOLCE], serpin family E member 1 [SERPINE1], and secreted protein acidic and rich in cysteine [SPARC]) (Fig. 4c). Gene ontology (GO) analysis of biological processes associated with the LSC secretome identified genes important in the developmental process, response to stimulus, and cellular component organization or biogenesis (Fig. 4d).

In addition, LSC and MSC exosomal proteins were also analyzed by LC/MS/MS for comparison (Supplementary Data 3 and 4, respectively). Interestingly, of the 102 common extracellular proteins identified in LSC-Sec approximately half were also identified in LSC-Exo (Fig. 4e). Gene ontology analysis showed many of the LSC exosomal proteins identified are involved in biological adhesion, cell adhesion, extracellular matrix organization, extracellular structure organization, and platelet degranulation (Fig. 4f and Supplementary Fig. 4a–d).

### Therapeutic effects of LSC exosomes

**Fig. 5:**
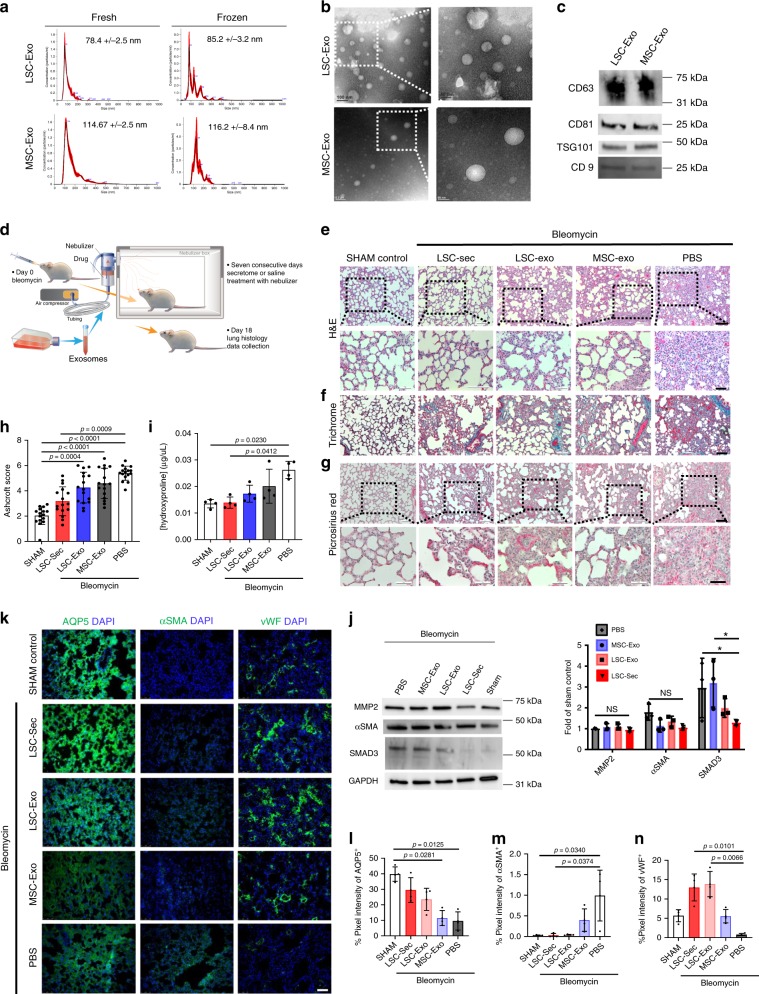
Therapeutic potential of exosome inhalation treatment in rats with pulmonary fibrosis. **a** Size analysis of fresh, frozen and lyophilized exosome particles by NanoSight. **b** Representative transmission electron micrograph (TEM) of LSC-Exo. Left scale bar = 0.2 μm. Right scale bar = 50 nm. **c** Immunoblot analysis of CD63, CD81, TSG101, and CD9 protein in LSC-Exo and MSC-Exo. **d** The experimental study schematic of the exosome study in SD rats; *n* = 12 biological independent animals per group. **e** Representative H&E staining Top: Scale bar = 100 μm Bottom: Scale bar = 50 μm. **f** Representative Gomori’s trichrome staining Scale bar = 100 μm (bottom); muscle fibers (red), collagen (blue), nuclei (black-purple) and erythrocytes (red). **g** Representative picrosirius red staining; Collagen types I and III (red); Scale bar = 50 μm. **h** Quantification of fibrosis by Ashcroft score; Each dot represents data from one animal; *n* = 12 biological independent animals. Ashcroft score was performed by averaging the score from one blinded and one non-blinded scorer. **i** Quantification of pulmonary hydroxyproline levels; *P ≤ 0.05; each dot represents data from one animal; *n* = 4 biological independent animals. **j** Immunoblot analysis of matrix metalloproteinase 2 (MMP-2), alpha smooth muscle actin (αSMA), SMAD3 and GAPDH loading control. **k** Representative immunostaining of lung sections for aquaporin 5 (AQP5), alpha-smooth muscle actin (αSMA), and von Willebrand Factor (vWF). Scale bar = 75 μm. **l**–**n** Quantification of pixel intensity of AQP5 (**l**), α-SMA (**m**), and vWF (**n**). Each dot represents data from one animal; *n* = 4 biological independent animals. Throughout, data are mean ± s.d. *P*-value as indicated by non-parametric one-way ANOVA.

We and others have demonstrated that the therapeutic components in secretome is comprised of not only soluble proteins but also extracellular vesicles (EVs), particularly exosomes (Exo). We characterized the LSC-Exo and MSC-Exo by size, morphology, and common exosomal markers (CD63, CD81, and TSG101) (Fig. 5a–c). For all in-vivo studies frozen exosomes were used; therefore, microRNA was analyzed to ensure that the contents from fresh to frozen exosomes are not altered (Supplementary Fig. 5a). Cellular uptake of exosomes was also verified (Supplementary Fig. 5b–c). To test the functional effects of exosomes, we employed a Bleo-induced PF model in SD rats similar to the mouse model previously presented (Fig. 5d)^21^. By utilizing rats in this study we were able to measure pulmonary function and evaluate the respiratory system mechanics using the forced oscillation techniques employed by the FlexiVent.

All secretome and exosome treated groups showed therapeutic effects in terms of maintaining normal lung architecture (Fig. 5e–f) and having decreased fibrosis (Fig. 5h), lung apoptosis (Supplementary Fig. 4a–d), and collagen deposition (Fig. 5g–i). Notably, only LSC-Sec and LSC-Exo significantly decreased collagen deposition, compared to the PBS treated group. In addition, only LSC-Sec significantly decreased fibrosis (Ashcroft score) to levels similar to the sham control. Lung protein levels of αSMA showed a non-significant trend of decline in treatment groups (Fig. 5j). LSC-Sec and LSC-Exo treatment attenuated alveolar epithelial and vascular injury, and reduced fibrotic response, as shown by increased AQP5^+^ and vWF^+^ cells, and decreased αSMA^+^ cells (Fig. 5k–n).

### Effects of LSC-Sec and LSC-Exo therapy on MMP-2 and MCP-1 expression

**Fig. 6:**
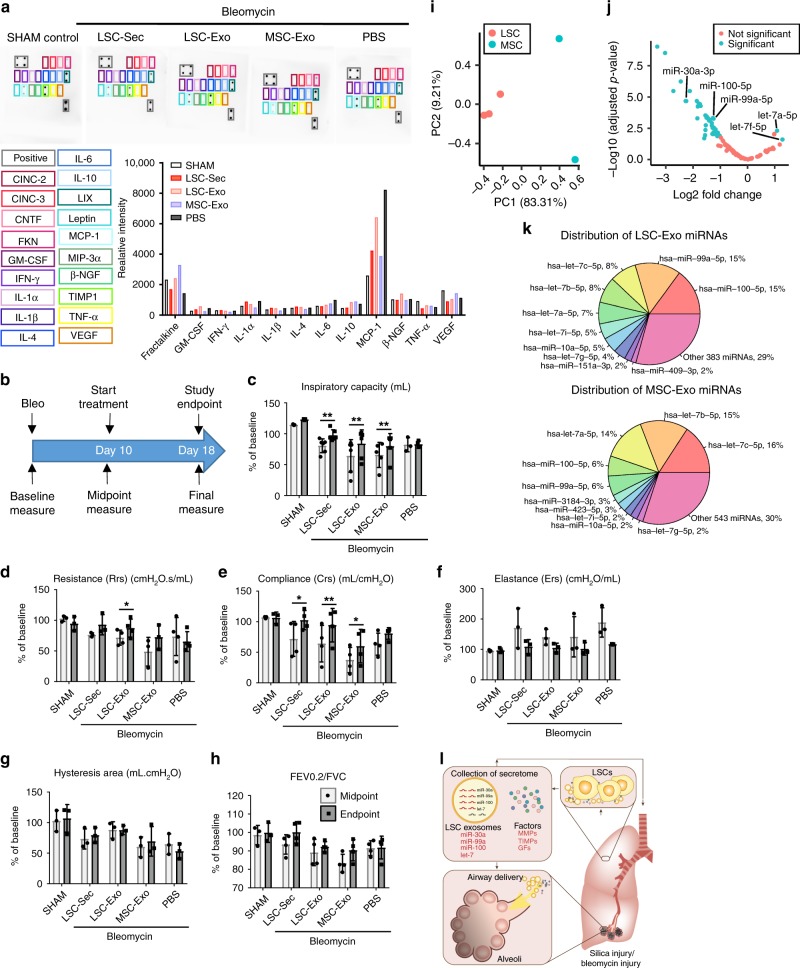
Exosome treatment improves pulmonary function post-Bleo and exosome miRNA profiling. **a** Representative rat cytokine array detecting 19 rat proteins from blood serum. **b** The schematic of pulmonary function measurements. **c**–**g** Quantification of lung function measurements (**c**) pulmonary inspiratory capacity; (**d**) pulmonary elastance (**e**) pulmonary resistance; (**f**) pulmonary compliance; (**g**) hysteresis area; (**h**) forced expiratory volume (FEV) to forced vital capacity (FVC) ratio. **i** Principal component analysis chart of LSC-exosome and MSC-exosome microRNA content. **j** Volcano plot of differentially expressed miRNA of LSC-exosomes and MSC-exosomes miRNA content. **k** Distribution of the top 10 miRNAs in LSC-exosomes (top) and MSC-exosomes (bottom). **l** LSC secreted exosomes and soluble factors modulate alveolar repair and fibrosis. Throughout, data are mean ± s.d. *P*-value as indicated by non-parametric one-way ANOVA (**a**) and Student’s paired *t*-test (**c**–**h**).

To examine potential molecular mechanisms involved in the response to secretome and exosome initiated responses, we examined systemic cytokine expression and lung tissue protein levels of MMP-2 and SMAD3. Similar to the murine Bleo model, here we found no significant change in MMP-2 protein levels in lungs of treated animals (Fig. 5j). However, SMAD3 levels in the lungs were significantly upregulated in the PBS group and were alleviated by LSC-Sec and LSC-Exo treatment. Because the immunomodulating effects of cytokines are systemic rather than local, we examined cytokine levels in the blood serum of treated animals (Fig. 6a). Monocyte chemoattractant protein-1 (MCP-1/CLL2) was found to be upregulated by Bleo injury, and showed a trending reduction by secretome and exosome treatment.

### Effects of secretome and exosome therapies on lung function

Next, we sought to predict the clinical impact of LSC-Sec and LSC-Exo treatment on impaired lung architecture and fibrosis. We, therefore, performed multiple pulmonary function tests in parallel with the inhalation treatment to monitor the decline in lung function following Bleo injury and any effects of the various treatments. Baseline reads were recorded immediately before Bleo instillation, midpoint reads were taken at day nine post-Bleo (the day before treatment started), and endpoint measurements were taken at day 17 post-Bleo (one day after the last inhalation treatment) (Fig. 6b). As expected, inspiratory capacity (IC), resistance (Rrs), compliance (Crs), hysteresis area, and the ratio of forced expiratory volume (FEV) to forced vital capacity (FVC) all showed a decline at midpoint compared to baseline (Fig. 6c–e, g, h). Consistently, respiratory elastance (Ers), which is the inverse of compliance, consistently increased at midpoint analysis (Fig. 6f). Altogether, pulmonary function of all Bleo animals was consistently impaired, as expected, with pulmonary injury, resulting in increased tissue stiffness and elastic recoil (elastance).

At endpoint analysis, pulmonary function was only partially rescued by secretome and exosome treatment. Inspiratory capacity and respiratory compliance were both significantly improved after LSC-Sec, LSC-Exo, or MSC-Exo treatment (Fig. 6c–e). Respiratory resistance had a significant recovery only with LSC-Exo treatment (Fig. 6d). Elastance, hysteresis area, and FEV/FVC ratio all had no significant changes after treatment.

### Toxicity of secretome and exosome therapies

In addition, to the effectiveness of secretome and exosome treatment, we also assessed the safety of the treatments. Liver and kidney function were found to be within an acceptable range in all treatment groups (Supplementary Fig. 7). Histological analysis of the heart, kidneys, liver, and spleens of treated animals in both the silica and Bleo studies did not reveal any apparent damage or toxicity (Supplementary Fig. 8). Animal survival and adverse effects were also monitored during all in-vivo studies (Supplementary Data 5).

### microRNA profiling of LSC and MSC exosome

Exosomes contain a variety of different RNAs, proteins, and lipids, but miRNAs have garnered significant interest since their discovery within exosomes^22^. It is believed that exosomes utilize miRNAs as a mechanism for genetic exchange between cells. We performed a global small RNA deep sequencing of LSC-Exo and MSC-Exo, and analyzed the differences in their miRNA compositions (Supplementary Fig. 9). Together, over 600 unique miRNAs were detected in these LSC-Exo and MSC-Exo samples, indicating that exosomal-derived miRNAs could have numerous regulatory roles in these samples. After removing less abundant miRNAs, 142 remained for downstream analysis (Fig. 6i, j, Supplementary Fig. 9b, and Supplementary Data 6). In total, 42 miRNAs were found to be differentially expressed between these two exosome types (minimum fold change of two and adjusted *p-*value < 0.05). Among the top upregulated miRNAs in LSC-Exo were hsa-miR-99a-5p (log2 fold change 1.25, *p* *=* 0.0005) and hsa-miR-100-5p (log2 fold change 1.27, *p* *=* 0.0005) (Fig. 6j, Supplementary Data 7). They were also the two most abundant miRNAs in LSC-Exo and are members of the miR-99 family (Fig. 6k). Notably, antifibrotic miR-30a-3p was also significantly upregulated in LSC-Exo compared to MSC-EXO (log2 fold change 2.28, *p* *=* 0.00002). hsa-let-7a-5p (log2 fold change 1.09, *p* *=* 0.001) and hsa-let-7f-5p (log2 fold change 1.28, *p* *=* 0.008) were the most upregulated in the MSC-Exo sample and are part of the highly conserved let-7 family. Sequence homology of human to rodent miRNAs was also examined since exosomes of human origin was utilized in the studies (Supplementary Data 8).

## Discussion

Various animal models have been developed to mimic the pathological hallmarks of human IPF^23,24^. Due to the unknown cause of the disease, a true IPF animal model does not exist. The Bleo model of induced pulmonary fibrosis is widely used for the study of IPF and is arguably the most clinically relevant^16,25^. To support any therapeutic effects found in the Bleo model, we also tested using the silica-induced pulmonary fibrosis model.

Both the Bleo and silica mouse models created significant alveolar epithelial damage and ECM deposition (Figs. 1g–k, 3c–g, and 5e–i). In the rat Bleo model, we also showed a significant decline in pulmonary function following Bleo injury (Fig. 6c–h). We and others have demonstrated that the cell-free secretome derived from stem cells can achieve a level of protection and regeneration similar or superior to the cells themselves^4,26–29^. Our observations showed that LSC-Sec was capable of reversing fibrosis caused by Bleo or silica particles (Figs. 1–3). However, the regenerative effects of LSC-Sec were more robust in the Bleo model than in the silica model. LSC-Sec inhalation in the Bleo-injured mice was able to rescue the damaged lungs back to levels similar to the healthy sham control in terms of decreased apoptotic cells, fibrotic score, hydroxyproline content, and significant recovery of AT1 and AT2 cell populations (Figs. 1c–h–k and 2a–d).

Secretome treatment in the silica model, did decrease fibrosis compared to the PBS group. However, it did not reverse the damage back to levels similar to healthy sham controls as seen in the Bleo studies. This could be due to the difference in the animal models. Silica injury is caused by the physical presence of particles deposited in the lung, which cause fibrotic nodules. Histological examination showed a decrease in fibrotic tissues surrounding the nodules in secretome treated groups, compared to the PBS control, but cell secretome therapy was not enough to resolve the nodules themselves (Fig. 3c–e, f). It is possible that seven days of treatment was not sufficient to reverse the damage caused by the silica particles. It would be beneficial to see if extended treatment would be more effective. For particulate-induced lung injuries, where matter is retained in the lungs, such as silicosis, the resolution of fibrosis may require not just an extended inhalation therapy, but also the clearing of the particles from the lung tissue. We plan to conduct additional studies utilizing higher doses of LSC-Sec and/or longer treatment duration to determine the optimal treatment conditions necessary to elicit a stronger therapeutic effect. It is possible that LSC-Sec alone is insufficient in resolving silica induced PF and additional therapies may be required.

The field of regenerative medicine utilizes many types of stem cells for research and clinical applications, but mesenchymal stem cells remain the most widely used, partly due to their immunomodulating abilities and ease of isolation^6,12,27^. Therefore, we wanted to compare the regenerative benefits of LSC-Sec against MSC-Sec. Our observations in both Bleo and silica models demonstrated that while MSC-Sec inhalation therapy was effective in treating pulmonary fibrosis, LSC-Sec was superior to MSC-Sec in all measures (Figs. 1–3).

We aimed to elucidate the molecular mechanism(s) underlying cell secretome-mediated lung repair through proteomic analyses. Such analyses revealed downregulation of αSMA (a myofibroblast marker) and pro-inflammatory/pro-fibrotic cytokines, such as IL-4, possibly through the upregulation of MMP-2 activity (Fig. 2e–h). Studies show that MMPs play diverse roles in PF, suggesting that in the early stages of Bleo-induced PF MMPs may contribute to disease pathogenesis^30,31^. However, MMP activity in the late stage may play a role in the repair process. It has been reported that MMPs play a pro-inflammatory/pro-fibrotic role in PF. However, it has also been shown that MMP activity is required for fibrosis resolution and our results supports the notion that in late stage fibrosis MMPs, including MMP-2, could be beneficial and can alter the course of fibrosis resolution^32^. MMPs not only play a role in pro-inflammatory and anti-inflammatory processes, but they also play a critical role in cell migration, proliferation, and angiogenesis by modifying the matrix environment^33–35^. MMP-2 null mice have been reported to be anatomically smaller than wild-type mice with less total vascular development, abnormal lung alveolarization and interestingly, decreased rates of tumor angiogenesis^36^. Mass spectrometry analysis of the LSC secretome revealed proteins related to the complement system (C1r and decorin), as well as Wnt signaling pathway (DKK3) (Fig. 4 and Supplementary Data 1). In addition, an abundance of ECM remodeling, pro-angiogenic, and cell proliferation proteins were also identified, suggesting that LSC-Sec and LSC-Exo contains proteins able to not only break down and reverse the fibrosis already in place, but also promote epithelial and vascular growth and repair (Fig. 4c–f, Supplementary Fig. 4, and Supplementary Data 1, 3).

Cell secretome does not merely contain soluble proteins, but also extracellular vesicles, such as exosomes. Exosomes are an emerging field of biomedical research that has seen an explosion of discoveries and interest in the last two decades. In fact, the therapeutic effects of stem cells have been attributed to the miRNAs found in secreted exosomes^6,22,37–39^. Therefore, we sought to determine if the regenerative benefits observed in response to LSC-Sec were due to exosomes. Our findings indicated that LSC-Exo were capable of reproducing part of the regenerative potency of the full secretome from which they were isolated (Figs. 5–6). We also demonstrated that LSC-Exo outperformed MSC-Exo in resolving pulmonary fibrosis and restoring healthy lung function. As expected, all secretome and exosome treated groups showed significant improvement in lung health. However, both LSC-Sec and LSC-Exo exhibited superior therapeutic effects compared to both MSC-Sec and MSC-Exo. LSC-Sec was the only treatment capable of reversing the alveolar damage, and reducing the fibrotic score and hydroxyproline levels to that of the sham control. Only LSC-Exo treatment induced significant recovery of total respiratory resistance (Fig. 6d).

RNA sequencing analysis of LSC-Exo and MSC-Exo showed the enrichment of miRNAs in the let-7 and miR-99 families, both present in the top ten miRNAs identified in both LSC-Exo and MSC-Exo (Fig. 6k). The let-7 family of miRNAs was the first miRNAs discovered and is highly conserved in plants and animals^40^. Let-7 miRNAs has been found to play an essential role in biological development, stem cell differentiation, and tumorigenesis. The miR-99 family (miR-99a, miR-99b, and miR-100) of miRNAs are also highly conserved miRNAs, highly expressed in stem cells, and are downregulated in lung injury and cancer^41,42^. Different subtypes of let-7 miRNAs are found in specific tissues, cells, and cancer types. Interestingly, let-7a, c, g, and miR-100 are downregulated in lung cancer, and all four are in the top 10 miRNAs detected in both LSC-Exo and MSC-Exo. miR-99a, miR-100, and miR-10a have also been found to be modulated in response to DNA damage and in lungs exposed to cigarette smoke^43^. The miR-99 family of microRNAs, along with miR-151a, miR-10a, and miR-30a, is significantly upregulated in LSC-Exo, compared to the other miRNAs identified. Likewise, let-7a and let-7f are significantly upregulated in MSC-Exo. Notably, miR-30a expression has been reported to be downregulated in IPF patients^44^. Studies have shown the antifibrotic properties of miR-30a in reversing transforming growth factor-beta (TGF-β)-induced Wnt1-inducible signaling pathway protein 1 (WISP1), inhibiting mitochondria fission, and preventing apoptosis. WISP1 is a known profibrotic mediator in IPF patients shown to enhance ECM deposition and promote fibrotic progression^45^. Even though miR-30a and the let-7 and miR-99 family of miRNAs are identified in both LSC-Exo and MSC-Exo, their differential expression may explain the difference in their treatment capacities.

To assess any systemic immunogenicity or off-target effects of inhaled cell secretome and exosome therapies, we examined the systemic cytokine expression, blood biochemistry, and histology of all major organs (Figs. 2g–h, 6a, and Supplementary Figs. 7–8). Normal tissue histology in all organs, along with normal liver and kidney functions, suggest that cell secretome and exosome treatment does not elicit any local or systemic immune reaction and does not cause any aberrant cellular or tissue alterations. Interestingly, our analysis of systemic cytokine expressions showed a trending downregulation of monocyte chemoattractant protein 1 (MCP-1, also known as chemokine ligand 2 [CCL2]) in the blood serum of treated animals (Fig. 6a). Profibrotic cytokine MCP-1 has been reported to play a key role in lung inflammation, and an increase in MCP-1 levels has been linked to poor prognosis for IPF patients^46,47^.

So far, secretome and exosomes from MSCs have been explored for biomarker and diagnostic applications^48,49^. Studies utilizing secretome and exosomes for therapeutic treatment are mainly administer by direct injection into the targeted tissue or systemically into a vein. Since we aim to treat the lungs, inhalation is the most direct and minimally invasive route of delivery. At our current dose of 10 mg of protein/kg of body weight it is feasible to generate that amount of secretome for clinical applications in humans using our current method. Nonetheless, future studies will be necessary for developing innovative methods (e.g., bioreactors) for the scale up of secretome and exosome production and isolation.

We acknowledge several limitations to this work. First, we used only one dose of cell secretome and exosomes, determined arbitrarily and guided by previous experience. It would be sensible for future studies to include a dose-response study to determine the least effective dosage and optimal dosage of both LSC-Sec and LSC-Exo. Secondly, our treatment in all three studies was delivered via inhalation using a nebulizer. An investigation into different administration routes to determine the optimal delivery method and frequency for LSC-Sec and LSC-Exo would help maximize their benefits. Thirdly, we isolated our exosomes by ultra-centrifugation and ultra-filtration using a 100 kDa MWCO ultrafiltration filter, which could allow the possibility of other extracellular vesicles and proteins larger than 100 kDa to also be isolated alongside the exosomes. Alternatively, we could have used a commercially available exosome isolation kit, however studies have shown that exosomes isolated via kits could potentially contain contamination from chemicals in the kits that could affect in-vivo applications and analysis^50,51^. Lastly, we presented here the proteomic and genomic composition of LSC-Sec and LSC-Exo, respectively, but it would be clinically and pharmaceutically beneficial to determine which proteins, small molecules, or RNAs are the functional components capable of achieving the therapeutic effects. Further biochemical and mechanistic characterization of LSC-Sec and LSC-Exo is required to completely elucidate the therapeutic function of LSC-Sec and LSC-Exo including identifying which factors are responsible for the effects observed, as well as which cells are up-taking the factors. The proteins and miRNAs reported here provide an initial insight into the therapeutic potential of LSC factors including proteins and secreted extracellular vesicles.

In summary, we report novel acellular therapeutic agents, namely LSC-Sec and LSC-Exo, shown to be safe and effective in the treatment of bleomycin-induced and silica-induced pulmonary fibrosis in rodents. The evidence presented here suggests that the mechanism of LSC-Sec mediated regeneration may relate to the exosomes, MMP-2 activity, and a host of proteins found in the cell secretome. Secretome and exosomes are considerably less immunogenic then their parent cells and the administration of these factors can overcome the limitations of stem cells while maintaining similar therapeutic effects. The identification of miR-30a and the miR-99 and let-7 family of miRNAs in LSC-Sec warrants future investigations, since miR-30a is known to be downregulated in IPF patients, and the miR-99 and let-7 families of miRNAs are also known to be downregulated in various cancers, including lung cancer. Idiopathic pulmonary fibrosis is currently an incurable respiratory disorder with increasing rates of morbidity and mortality; with no effective therapies currently available, LSC-Sec and LSC-Exo provide promising candidates for the development of IPF therapies.

## Methods

### Cell culture

Human LSCs were generated from whole lung samples and expanded as described^10–12^. Human LSCs were generated from healthy whole lung donors acquired from the Cystic Fibrosis and Pulmonary Diseases Research and Treatment Center at the University of North Carolina at Chapel Hill and expanded as described in Fig. 1a. Human bone marrow-derived MSCs were obtained from the American Type Culture Collection (ATCC). We used passage 2–5 LSCs and MSCs for all in-vitro and in-vivo testing. Cells were analyzed by flow cytometry for the appropriate markers before use (for LSCs: CD90^+^, CD105^+^, CCSP^+^, AQP5^+^, ProSPC^+^, Epcam^+^, CD31^−^, CD34^−^, and CD45^−^ and for MSCs: CD90^+^, CD105^+^, CD31^−^, CD34^−^, and CD45^−^). Healthy human lung tissues were acquired from the Cystic Fibrosis and Pulmonary Diseases Research and Treatment Center at the University of North Carolina-Chapel Hill. All procedures performed in this study involving human samples were in accordance with the ethical standards of the institutional research committee and with the guidelines set by the Declaration of Helsinki.

### Secretome collection and preparation

Human LSCs and MSCs were cultured to approximately 75% confluence before the serum-containing media was removed and replaced with serum-free media (IMDM). The following day, the cells were washed six times for 30 min each with fresh IMDM to remove serum albumin from the cells before the media was allowed to condition. Albumin can interfere with some experiments (especially LC/MS/MS analysis). The media (IMDM) was allowed to condition for three days before it was harvested and filtered through a 0.22 μm filter to remove any cells and cell debris. The filtered secretome was stored at −80 °C for at least 24 h or until solid. The frozen secretome vial(s) were lyophilized overnight or until samples were dehydrated, using a LABCONCO FreeZone 2.5 L Freeze Dry System. Once samples were lyophilized, they were stored at −20 °C until ready to use.

### Exosome isolation and characterization

Exosomes were collected and purified from human LSC-Sec and MSC-Sec using an ultrafiltration method^52^. The secretomes were first filtered through a 0.22 μm filter to remove any cells or cell debris. The filtered secretomes were then placed in a 100 kDa MWCO ultrafiltration filter (Millipore) and centrifuged at 5000 × *g* for 10–15 min depending on volume. Any media contents or small proteins were removed by the filtered centrifugation, and the remaining exosomes were suspended in PBS, then filtered and washed. Before use, all exosome samples were analyzed for proper size by nanoparticle tracking analysis (NTA; NanoSight, Malvern), and for morphology by transmission electron microscopy (TEM). In addition, successful exosome isolation was confirmed by immunoblotting for known exosome markers CD63 (NB100-77913, Novus), CD81 (SAB3500454, Sigma-Aldrich), and TSG101(MA1-23296, Thermo Fisher).

### Animal procedures

Six to eight week old male CD1 mice [Crl: CD1(ICR)] and CD (SD) IGS rats [Crl: CD(SD)] were obtained from Charles River Laboratory (Massachusetts, USA), and A/J mice were obtained from Jackson Laboratory (Maine, USA). Pulmonary fibrosis was induced with a single intratracheal injection of 3 U/kg bleomycin sulfate (EMD; 203401) solution in CD1 mice and CD (SD) rats and a single oropharyngeal aspiration of a 100 mg/kg silica (MIN-U-SIL-5) suspension in A/J mice. Nebulizer treatment started 10 days post bleomycin insult and 28 days post silica insult. Secretome, exosome, or saline inhalation treatment was given for approximately 30 min/day for seven consecutive days using a nebulizer (Pari Trek S Portable Compressor Nebulizer Aerosol System; 047F45-LCS). LSC and MSC secretome dose is standardized by protein concentration of 10 mg of secretome protein per kg of body weight. LSC and MSC exosome dose is standardized by the number of exosome particles (10 × 10^9^ particles per kg of body weight). Animals were euthanized, and blood and tissues were collected for RNA, protein, and histological examination. All studies and protocols were approved by the Institutional Animal Care and Use Committee at North Carolina State University.

### Pulmonary function test (PFT) in rats

Pulmonary function measurements were performed on the FlexiVent (SCIREQ Inc., Montreal, Canada). Prior to measurements, animals were anesthetized with an intraperitoneal injection of ketamine and xylazine solution (2:1 ratio). The animals were intubated with a 14-gauge cannula. Pulmonary function data is only reported for animals that has all three baseline, midpoint and endpoint measurement for analysis.

### Histology

Immunostaining was performed on tissue slides fixed in 4% paraformaldehyde (PFA) (Electron Microscopy Sciences; 15710) followed by permeabilization and blocking with Dako Protein blocking solution (DAKO; X0909) containing 0.1% saponin (Sigma-Aldrich; 47036) prior to antibody staining. Cells were stained at a dilution of 1:200 with antibodies against AQP5 (ab78486, Abcam), ProSPC (ab90716, Abcam), vWF (F3520, Sigma-Aldrich), αSMA (ab5694, Abcam), vimentin (ab20346, Abcam), CD105 (ab44967, Abcam), EpCAM (324204, Biolegend), Uteroglobin (ab140663, Abcam). Caspase 3 (ab2302, Abcam), and PARP (44-698 G, ThermoFisher). Tunel staining was performed on cryosectioned tissues using the In-Situ Cell Death Detection Kit (12156792910, Roche Diagnostics, Mannheim, Germany) according to the manufacturer’s instructions. Gomori’s Trichrome, and Hematoxylin and eosin were performed on paraffin embedded tissue sections. Hematoxylin and eosin stained sections were used for Ashcroft scoring. Ashcroft score was performed by averaging the scores from one blinded and one non-blinded scorer.

### Proteomic analysis

Secretome samples were prepared as follows. To concentrate the samples, 15 ml of the secretome was lyophilized. The samples dried to completion in 24 h and were re-suspended in 1 ml of 100 mM ammonium bicarbonate (Sigma Aldrich) buffer at pH 8.6. For protein precipitation, 10 ml of cold acetone (Optima grade, ThermoFisher Scientific) was added to the samples, incubated at −20 °C overnight, and then centrifuged for 30 min at 8176 × *g* to separate the precipitated proteins from the supernatant. Total protein concentration was determined using the Bradford Assay (Pierce, ThermoFisher Scientific). All samples were loaded in triplicate (10 μl) onto a microtiter plate, measured at an absorbance of 595 nm using a Tecan Genios microplate reader, and compared to the reference absorbance of BSA standard protein. Precipitated proteins were digested using the Filter Aided Sample Preparation (FASP) method as described by Wisniewski et al.^53^. In a 30 kDa molecular weight cutoff Vivacon filter (Satortorius, ThemoFisher Scientific), approximately 100 μg of protein from each sample were added, reduced with 5 mM dithiothereitol (ThermoFisher Scientific) at 56 °C for 30 min, and then alkylated with 10 mM iodoacetamide (Sigma-Aldrich) for 20 min in the dark at RT. Digestion on-filter was carried out using sequencing grade trypsin (Promega) at a 1:100 trypsin-to-protein ratio overnight at 37 °C. Peptides were removed from the filter with 100 mM ammonium bicarbonate buffer, pH 8.6, and the solvent was evaporated using vacuum centrifugation before storage at −20 °C for further processing.

LC/MS/MS analysis for all samples was performed on an Easy nano ultra-high pressure liquid chromatograph coupled to an LTQ Orbitrap Elite mass spectrometer (ThermoFisher Scientific). Samples were injected onto a PepMap C18, 5 μm trapping column (ThermoFisher Scientific), then separated by in-line gradient onto a New Objective Self Pack PicoFrit column (packed in house with 3.0 μm Reprosil C18 stationary phase). The linear gradient for separation was 5–40% mobile phase B over 90 min at 300 nl/min flow rate, where mobile phase A was 2% acetonitrile/0.1% formic acid in water, and mobile phase B was 0.1% formic acid in acetonitrile. The Orbitrap Elite operated in a data-dependent mode, where the five most intense precursors were selected for subsequent fragmentation. Resolution for the precursor scan (*m/z* 400–2000) was set to 60,000 at *m/z* 400 with a target value of 1 × 10^6^ ions. The MS/MS scans were also acquired in the orbitrap with a normalized collision energy setting of 27 for HCD. For internal mass calibration, the ion of polycyclodimethylsiloxane with *m/z* 445.120025 was used as the lock mass^54^. Monoisotopic precursor selection was enabled, and precursors with unknown charge or a charge state of +1 were excluded.

Raw data files were processed using Proteome Discoverer (1.4, ThermoFisher Scientific). Peak lists were searched against a forward and reverse Homo sapiens UniProt database using Mascot (1.4.1.14 Matrix Science, www.matrixscience.com). The parameters used for identification of tryptic peptides were: 10 ppm precursor ion mass tolerance, 0.01 Da fragment mass tolerance; up to two missed cleavage sites; carbamidomethylation of Cys was set as a fixed modification; oxidation of Met was set as a variable modification. Scaffold (4.8.4 Proteome software, www.proteomesoftware.com) was used to filter the data, quantify peptides/proteins, and perform statistical analysis. A minimum of 2 peptides per protein, at a peptide and protein threshold of 95%, were required for high confidence identification. Ingenuity Proteomic Analysis (IPA, QIAGEN Redwood City, www.qiagen.com/ingenuity) was used for classification of subcellular localization of the common proteins. The listed common secreted proteins were classified using Panther (Protein Analysis Through Evolutionary Relationships, htpp://pantherdb.org) and DAVID (The Database for Annotation, Visualization and Integrated Discovery, https://david.ncifcrf.gov) to explore molecular function, cellular components, and pathways.

Exosome samples were prepared as follows. To concentrate the samples for proteomic analysis exosome samples were lysed with 1× RIPA buffer then sonicated three times for 5 mins each. Exosome protein lysate was then concentrated with a 3 kDa MWCO ultrafiltration filter (Millipore) and centrifuged at 5000×*g* for 10–20 min depending on volume. For the LSC and MSC samples, 40 μg and 25 μg of protein from LSC and MSC exosome samples, respectively, were processed using FASP as described above for the secretome samples. LC/MS/MS analysis of the exosome samples was performed on an Easy nano ultra-high pressure liquid chromatograph coupled to a Q Exactive HF-X Hybrid Quadrupole-Orbitrap mass spectrometer (ThermoFisher Scientific). Samples were injected onto a PepMap C18, 3 μm trapping column (ThermoFisher Scientific) then separated by in-line gradient onto an EASY-Spray capillary column (75 μm × 25 cm packed with 2 μm C18 stationary phase) (ThermoFisher Scientific) using the same gradient as described above for the secretome samples. The Q Exactive HF-X Orbitrap was operated as described above for the LTQ Orbitrap Elite except for the following settings that are more appropriate for the HF-X system: resolution for the precursor scan (*m/z* 400–2000) was set to 120,000 at *m/z* 400 with a target value of 3 × 10^6^ ions, the resolution of the product ion scan was set at 7500, and the data-dependent acquisition mode selected the forty most intense precursors for subsequent fragmentation.

Raw data files were processed using Proteome Discoverer (2.2, ThermoFisher Scientific). Peak lists were searched against a Homo sapiens UniProt database using Sequest. The parameters used for identification of tryptic peptides were: 5 ppm precursor ion mass tolerance, 0.02 Da fragment mass tolerance; up to three missed cleavage sites; carbamidomethylation of Cys and oxidation of Met was set as a variable modifications; acetylation at N-terminus was set as a variable modification. Proteome Discoverer was used to filter the data, quantify peptides/proteins, and perform statistical analysis. A minimum of 2 peptides per protein at a peptide and protein threshold of 95% were required for high confidence identification. The listed common secreted proteins were classified using Panther (Protein Analysis Through Evolutionary Relationships, http://pantherdb.org) to explore molecular function, cellular components and pathways.

### SDS-PAGE and western blot

Samples were reduced by β-mercaptoethanol and denatured at 90 °C for 5 min. Proteins were separated by gel electrophoresis carried out in triplicate on a 4–15% Tris-Glycine stain-free gel (Bio-Rad), along with a molecular weight standard (Bio-Rad, Precision Plus Protein Unstained Standards MW Ladder 161-0363). The gel was run at a stack voltage at 100 V for approximately 5 min, followed by a constant 200 V. The gel was activated and visualized by UV light in a Bio-Rad Imager.

The Bio-Rad Mini-PROTEAN Tetra Cell system was used for a wet transfer. The SDS-PAGE gel was assembled into the apparatus with a PVDF membrane stacked between filter papers. Following the transfer, the membrane was washed three times in PBS-T for 5 min each, then blocked using 5% milk in PBS-T for 1 h. A 4 °C overnight incubation of the primary antibody was performed for MMP-2 (ab86607, Abcam), αSMA (ab5694, Abcam), SMAD3 (MA5-14939, Thermo Fisher Scientific), CD63 (nb100-77913, Novus Biologicals), CD81 (MA5-13548, ThermoFisher Scientific), CD9 (MA5-31980, ThermoFisher Scientific), beta-actin (MA5-15739, ThermoFisher Scientific) and GAPDH (MA5-15738-HRP, ThermoFisher Scientific) at a 1:1000 dilution. This was then followed by a 1-h incubation with the corresponding HRP conjugated secondary antibodies at a 1:10,000 dilution.

### Small RNA library construction and sequencing

Exosomal RNA was isolated using a commercially available total exosome RNA isolation kit (Qiagen’s exoRNeasy Serum Plasma Kit). Once exosomal RNA was isolated, it was further analyzed by sequencing. RNA quantity, 260/280 ratios, and 260/230 ratios, were assessed by Nanodrop (ThermoFisher). RNA integrity was verified on a Bioanalyzer 2100 using an Agilent RNA 6000 Nano Assay. Small RNA libraries were prepared according to the manufacturer’s protocol using a NEBNext® Multiplex Small RNA Library Prep Set for Illumina (New England Biolabs). Libraries were size selected using a Pippen Prep (Sage Science), and quality-checked by Bioanalyzer using an Agilent High Sensitivity DNA Kit. Libraries were quantified by a Quant-iT™ dsDNA High Sensitivity Assay Kit (ThermoFisher) and sequenced on an Illumina NextSeq500 using a mid-output V2 kit.

### Mapping and differential expression analysis of miRNAs

Raw reads were demultiplexed and quality trimmed using the standard Illumina bcl2fastq conversion software. Using mirDeep2, reads had adapters removed and were clustered using default parameters^55^. Reads were then aligned to human rRNAs using Bowtie, and the remaining unaligned reads were mapped to miRBase 22, also using Bowtie^56,57^. For downstream analysis, identified miRNAs were filtered by requiring that an miRNA have at least ten read counts in at least two of the five samples. Differential expression analysis between MSC samples and LSC samples was performed using the edgeR and limma R packages and upper quantile normalized read counts^58,59^. miRNAs were considered differentially expressed if the absolute log2 fold change was greater than 1 and if the adjusted p-value (FDR) was less than 0.05.

### Blood biochemistry

All blood biochemistry test was conducted by North Carolina State University Veterinary Hospital’s clinical pathology lab. All analysis was performed according to the International Federation of Clinical Chemistry and Laboratory Medicine (IFCC) on the Cobas C501 instrument by Roche. Alanine aminotransferase (ALT) and aspartate aminotransferase (AST) was determine by IFCC methods without pyridoxal-5-phosphate (P5P). Blood urea nitrogen (BUN) was determined by Urease/Glutamate dehydrogenase (GLDH). Creatinine was determined by the compensated kinetic Jaffe’s method.

### Statistical analysis

All results are expressed as the mean ± standard deviation (SD). Two-tailed paired Student’s t-test was used for comparison between two groups (Fig. 6c–h). Non-parametric one-way ANOVA (Kruskal-Wallis test) was used for comparison of three or more groups, with additional Bonferroni post hoc correction. Differences were considered statistically significant at *P* ≤ 0.05. *P ≤ 0.05; **P ≤ 0.01.

### Reporting summary

Further information on research design is available in the Nature Research Reporting Summary linked to this article.

## Supplementary information

Supplementary Information

Description of Additional Supplementary Files

Supplementary Data 1

Supplementary Data 2

Supplementary Data 3

Supplementary Data 4

Supplementary Data 5

Supplementary Data 6

Supplementary Data 7

Supplementary Data 8

Reporting Summary

## Data Availability

The data that support the findings of this study are available from the corresponding author upon reasonable request. Genomic data has been submitted to GEO with accession ID: GSE136263. Proteomic data has been submitted to Massive 10.25345/C5MM4T [ftp://massive.ucsd.edu/MSV000084596/]. Source data for all figures are provided as a Source Data file.

## Source Data

Source Data

## References

[1] Henry Eric, Cores Jhon, Hensley M. Taylor, Anthony Shirena, Vandergriff Adam, de Andrade James B.M., Allen Tyler, Caranasos Thomas G., Lobo Leonard J., Cheng Ke (2015). Adult Lung Spheroid Cells Contain Progenitor Cells and Mediate Regeneration in Rodents With Bleomycin-Induced Pulmonary Fibrosis. STEM CELLS Translational Medicine.

[2] Dinh, P. C. et al. Derivation of therapeutic lung spheroid cells from minimally invasive transbronchial pulmonary biopsies. *Respir. Res.* **18**, 132 (2017). 10.1186/s12931-017-0611-0 PMC5493087 28666430

[3] Cores J (2017). Safety and efficacy of allogeneic lung spheroid cells in a mismatched rat model of pulmonary fibrosis. Stem Cells Transl. Med..

[4] Yang, H. et al. Neural stem cell-conditioned medium ameliorated cerebral ischemia-reperfusion injury in rats. *Stem Cells Int*. **2018**, 4659159 (2018). 10.1155/2018/4659159 PMC5903322 29765412

[5] Bai L (2012). Hepatocyte growth factor mediates mesenchymal stem cell-induced recovery in multiple sclerosis models. Nat. Neurosci..

[6] Willis GR (2018). Mesenchymal stromal cell exosomes ameliorate experimental bronchopulmonary dysplasia and restore lung function through macrophage immunomodulation. Am. J. Respir. Crit. Care Med..

[7] Hutchinson JP, McKeever TM, Fogarty AW, Navaratnam V, Hubbard RB (2014). Increasing global mortality from idiopathic pulmonary fibrosis in the twenty-first century. Ann. Am. Thorac. Soc..

[8] Hamman L, Rich AR (1935). Fulminating diffuse interstitial fibrosis of the lungs. Trans. Am. Clin. Climatological Assoc..

[9] Homolka J (1987). Idiopathic pulmonary fibrosis: a historical review. Can. Med. Assoc. J..

[10] Raghu G, Johnson WC, Lockhart D, Mageto Y (1999). Treatment of idiopathic pulmonary fibrosis with a new antifibrotic agent, pirfenidone: results of a prospective, open-label Phase II study. Am. J. Respir. Crit. Care Med..

[11] Myllarniemi, M. & Kaarteenaho, R. Pharmacological treatment of idiopathic pulmonary fibrosis-preclinical and clinical studies of pirfenidone, nintedanib, and N-acetylcysteine. *Eur. Clin. Respir. J.* **2**, 26385 (2015). 10.3402/ecrj.v2.26385 PMC4629756 26557253

[12] Tzouvelekis A (2013). A prospective, non-randomized, no placebo-controlled, phase Ib clinical trial to study the safety of the adipose derived stromal cells-stromal vascular fraction in idiopathic pulmonary fibrosis. J. Transl. Med..

[13] Glassberg MK (2017). Allogeneic human mesenchymal stem cells in patients with idiopathic pulmonary fibrosis via intravenous delivery (AETHER): a phase I safety clinical trial. Chest.

[14] Ratajczak MZ (2011). Pivotal role of paracrine effects in stem cell therapies in regenerative medicine: can we translate stem cell-secreted paracrine factors and microvesicles into better therapeutic strategies?. Leukemia.

[15] Baraniak PR, McDevitt TC (2010). Stem cell paracrine actions and tissue regeneration. Regen. Med..

[16] Moeller A, Ask K, Warburton D, Gauldie J, Kolb M (2008). The bleomycin animal model: a useful tool to investigate treatment options for idiopathic pulmonary fibrosis?. Int. J. Biochem. Cell Biol..

[17] Huaux F, Liu T, McGarry B, Ullenbruch M, Phan SH (2003). Dual roles of IL-4 in lung injury and fibrosis. J. Immunol..

[18] Groves AM, Johnston CJ, Misra RS, Williams JP, Finkelstein JN (2016). Effects of IL-4 on pulmonary fibrosis and the accumulation and phenotype of macrophage subpopulations following thoracic irradiation. Int. J. Radiat. Biol..

[19] Zhang L (2018). Macrophages: friend or foe in idiopathic pulmonary fibrosis?. Respir. Res..

[20] McNeill DA, Chrisp CE, Fisher GL (1990). Pulmonary adenomas in A/J mice treated with silica. Drug Chem. Toxicol..

[21] Rathinasabapathy A (2016). Therapeutic potential of adipose stem cell-derived conditioned medium against pulmonary hypertension and lung fibrosis. Br. J. Pharmacol..

[22] Ratajczak J (2006). Embryonic stem cell-derived microvesicles reprogram hematopoietic progenitors: evidence for horizontal transfer of mRNA and protein delivery. Leukemia.

[23] Degryse AL, Lawson WE (2011). Progress toward improving animal models for idiopathic pulmonary fibrosis. Am. J. Med. Sci..

[24] Jenkins RG (2017). An official american thoracic society workshop report: use of animal models for the preclinical assessment of potential therapies for pulmonary fibrosis. Am. J. Respir. Cell Mol. Biol..

[25] Luna MA, Bedrossian CW, Lichtiger B, Salem PA (1972). Interstitial pneumonitis associated with bleomycin therapy. Am. J. Clin. Pathol..

[26] Tang J (2017). Therapeutic microparticles functionalized with biomimetic cardiac stem cell membranes and secretome. Nat. Commun..

[27] Luo L (2017). Fabrication of synthetic mesenchymal stem cells for the treatment of acute myocardial infarction in mice. Circ. Res..

[28] Tang J-N (2018). Concise review: is cardiac cell therapy dead? embarrassing trial outcomes and new directions for the future. Stem Cells Transl. Med..

[29] Hu S, Ogle BM, Cheng K (2018). Body builder: from synthetic cells to engineered tissues. Curr. Opin. Cell Biol..

[30] Kim JY, Choeng HC, Ahn C, Cho S-H (2009). Early and late changes of MMP-2 and MMP-9 in bleomycin-induced pulmonary fibrosis. Yonsei Med. J..

[31] Giannandrea M, Parks WC (2014). Diverse functions of matrix metalloproteinases during fibrosis. Dis. Model. Mech..

[32] Redente, E. F., Black, B. P., Edelman, B. L. & Riches, D. W. H. The resolution of pulmonary fibrosis is dependent upon matrix metalloproteinase-9 expression. In *B96 Complex Cell Behaviors in Pulmonary Fibrosis* A4021–A4021 (American Thoracic Society, 2019).

[33] van Hinsbergh VWM, Koolwijk P (2007). Endothelial sprouting and angiogenesis: matrix metalloproteinases in the lead. Cardiovasc. Res..

[34] Ray JM, Stetler-Stevenson WG (1994). The role of matrix metalloproteases and their inhibitors in tumour invasion, metastasis and angiogenesis. Eur. Respir. J..

[35] Itoh T (1998). Reduced angiogenesis and tumor progression in gelatinase a-deficient mice. Cancer Res..

[36] Kheradmand F, Rishi K, Werb Z (2002). Signaling through the EGF receptor controls lung morphogenesis in part by regulating MT1-MMP-mediated activation of gelatinase A/MMP2. J. Cell Sci..

[37] Vandergriff AC (2015). Intravenous cardiac stem cell-derived exosomes ameliorate cardiac dysfunction in doxorubicin induced dilated cardiomyopathy. Stem Cells Int..

[38] Vandergriff A (2018). Targeting regenerative exosomes to myocardial infarction using cardiac homing peptide. Theranostics.

[39] Théry, C., Curie, A. I. & Inserm, U. Exosomes: secreted vesicles and intercellular communications. *F1000 Biol. Rep.* **3**, 15 (2011). 10.3410/B3-15 PMC3155154 21876726

[40] Johnson SM (2005). RAS is regulated by the let-7 MicroRNA family. Cell.

[41] Mueller AC, Sun D, Dutta A (2012). The miR-99 family regulates the DNA damage response through its target SNF2H. Oncogene.

[42] Qin X (2017). Cisplatin-resistant lung cancer cell–derived exosomes increase cisplatin resistance of recipient cells in exosomal miR-100–5p-dependent manner. Int. J. Nanomed..

[43] Izzotti A (2018). Downregulation of microRNA expression in the lungs of rats exposed to cigarette smoke. FASEB J..

[44] Pandit KV, Milosevic J, Kaminski N (2011). MicroRNAs in idiopathic pulmonary fibrosis. Transl. Res..

[45] Konigshoff M (2009). WNT1-inducible signaling protein-1 mediates pulmonary fibrosis in mice and is upregulated in humans with idiopathic pulmonary fibrosis. J. Clin. Invest..

[46] Car BD (1994). Elevated IL-8 and MCP-1 in the bronchoalveolar lavage fluid of patients with idiopathic pulmonary fibrosis and pulmonary sarcoidosis. Am. J. Respir. Crit. Care Med..

[47] Antoniades HN (1992). Expression of monocyte chemoattractant protein 1 mRNA in human idiopathic pulmonary fibrosis. Proc. Natl Acad. Sci. USA.

[48] Jia Y (2017). Exosome: emerging biomarker in breast cancer. Oncotarget.

[49] Schey KL, Luther JM, Rose KL (2015). Proteomics characterization of exosome cargo. Methods.

[50] Patel GK (2019). Comparative analysis of exosome isolation methods using culture supernatant for optimum yield, purity and downstream applications. Sci. Rep..

[51] Skottvoll Frøydis Sved, Berg Henriette Engen, Bjørseth Kamilla, Lund Kaja, Roos Norbert, Bekhradnia Sara, Thiede Bernd, Sandberg Cecilie, Vik-Mo Einar Osland, Roberg-Larsen Hanne, Nyström Bo, Lundanes Elsa, Wilson Steven Ray (2019). Ultracentrifugation versus kit exosome isolation: nanoLC–MS and other tools reveal similar performance biomarkers, but also contaminations. Future Science OA.

[52] Greening DW, Xu R, Ji H, Tauro BJ, Simpson RJ (2015). A protocol for exosome isolation and characterization: evaluation of ultracentrifugation, density-gradient separation, and immunoaffinity capture methods. Methods Mol. Biol..

[53] Wisniewski JR, Zougman A, Nagaraj N, Mann M (2009). Universal sample preparation method for proteome analysis. Nat. Methods.

[54] Olsen JV (2005). Parts per million mass accuracy on an Orbitrap mass spectrometer via lock mass injection into a C-trap. Mol. Cell. Proteom..

[55] Friedländer MR, Mackowiak SD, Li N, Chen W, Rajewsky N (2012). miRDeep2 accurately identifies known and hundreds of novel microRNA genes in seven animal clades. Nucleic Acids Res..

[56] Langmead B, Trapnell C, Pop M, Salzberg SL (2009). Ultrafast and memory-efficient alignment of short DNA sequences to the human genome. Genome Biol..

[57] Kozomara A, Griffiths-Jones S (2014). miRBase: annotating high confidence microRNAs using deep sequencing data. Nucleic Acids Res..

[58] Robinson MD, McCarthy DJ, Smyth G (2010). K. edgeR: a Bioconductor package for differential expression analysis of digital gene expression data. Bioinformatics.

[59] Ritchie ME (2015). limma powers differential expression analyses for RNA-sequencing and microarray studies. Nucleic Acids Res..

