# Supplementary material for: Inhalation of lung spheroid cell secretome and exosomes promotes lung repair in pulmonary fibrosis
Source: Nat Commun. 2020 Feb 28;11:1064. doi: 10.1038/s41467-020-14344-7 (PMC7048814; doi:10.1038/s41467-020-14344-7)
Supplement: Supplementary file 1 — Supplementary Information [file 41467_2020_14344_MOESM1_ESM.pdf]

Supplementary Information

Lung Spheroid Cell-Secreted Factors Promotes Alveolar Repair and Attenuates Pulmonary Fibrosis

Dinh et al.

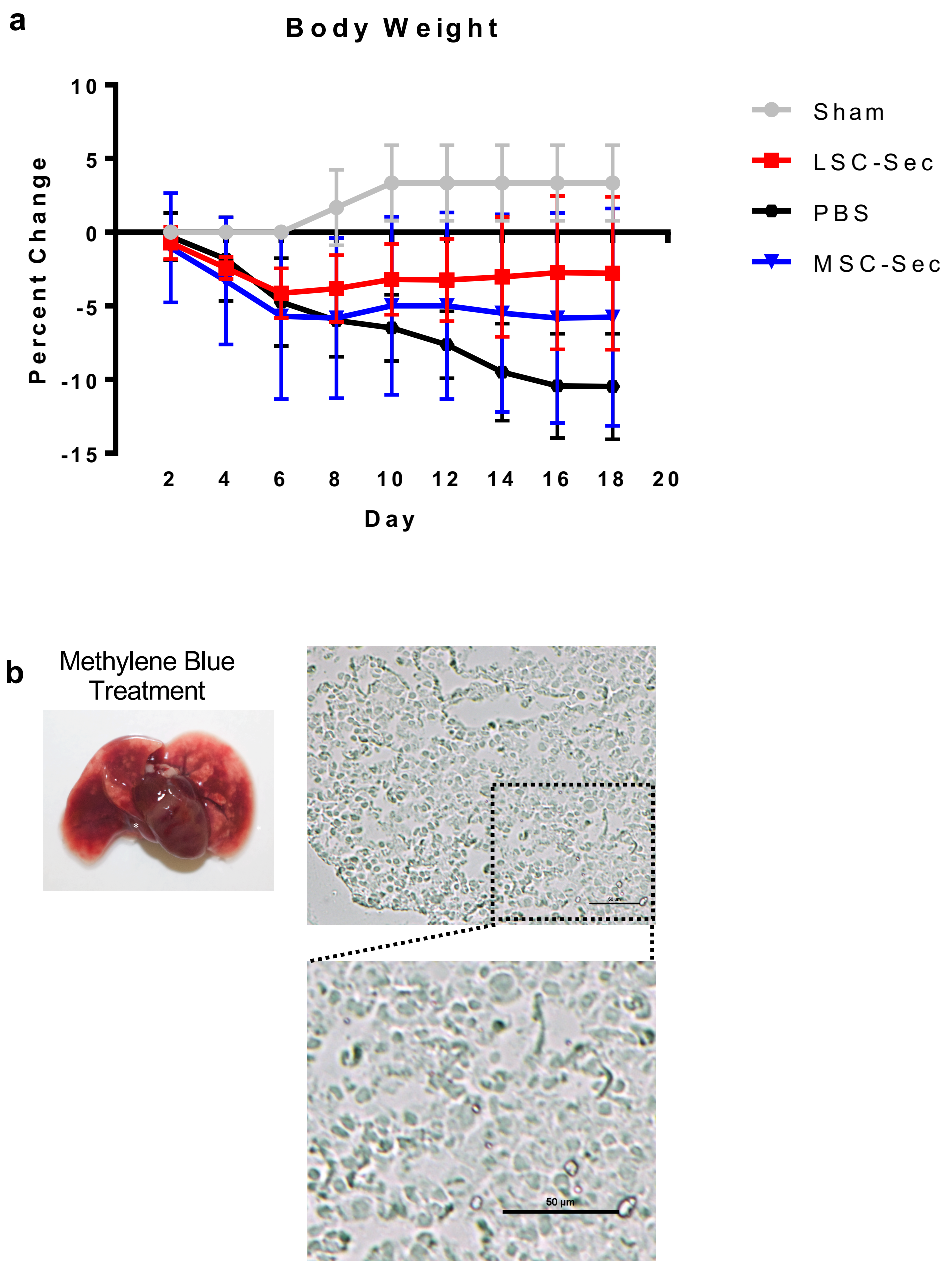

Supplementary Figure 1. Preliminary proof of concept for inhalation treatment of bleomycin pulmonary fibrosis model. (**a**) CD1 mice body weight measures throughout the study. (**b**) Methylene blue nebulization experiment in CD1 mice. Left: explanted mice lung and heart. Right: Unstained cryosectioned lung tissue. Scale bar= 50 μm.

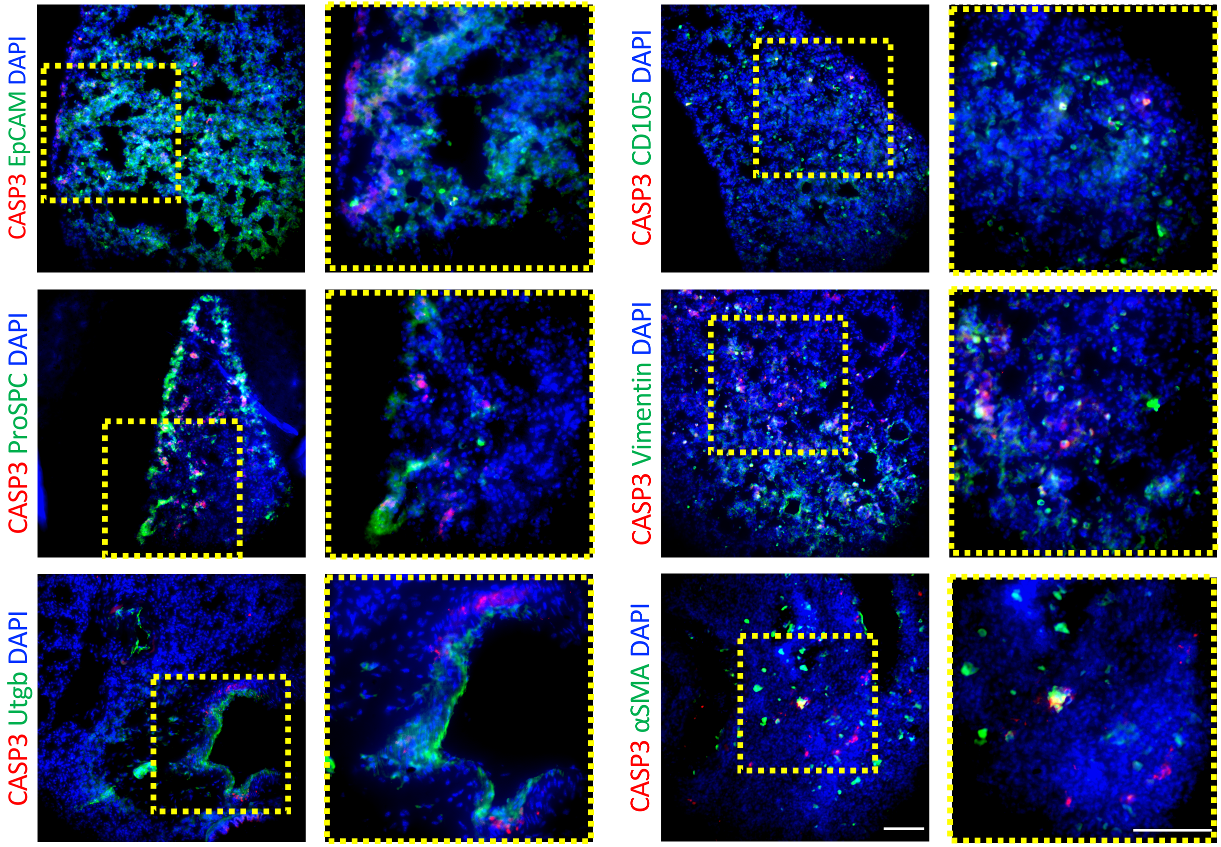

Supplementary Figure 2. LSC-secretome study immunostained analysis of apoptotic cells. Representative Caspase 3 immunostaining of apoptotic cells co-stained with epithelial cell adhesion molecule (EpCAM), prosurfactant protein C (Pro-SPC/Sftpc), uteroglobin (Utgb/Scgb1a1), CD105, vime ntin, and alpha smooth muscle actin (αSMA) in lung tissues of mice post-bleomycin lung injury. Scale bars= 100μm

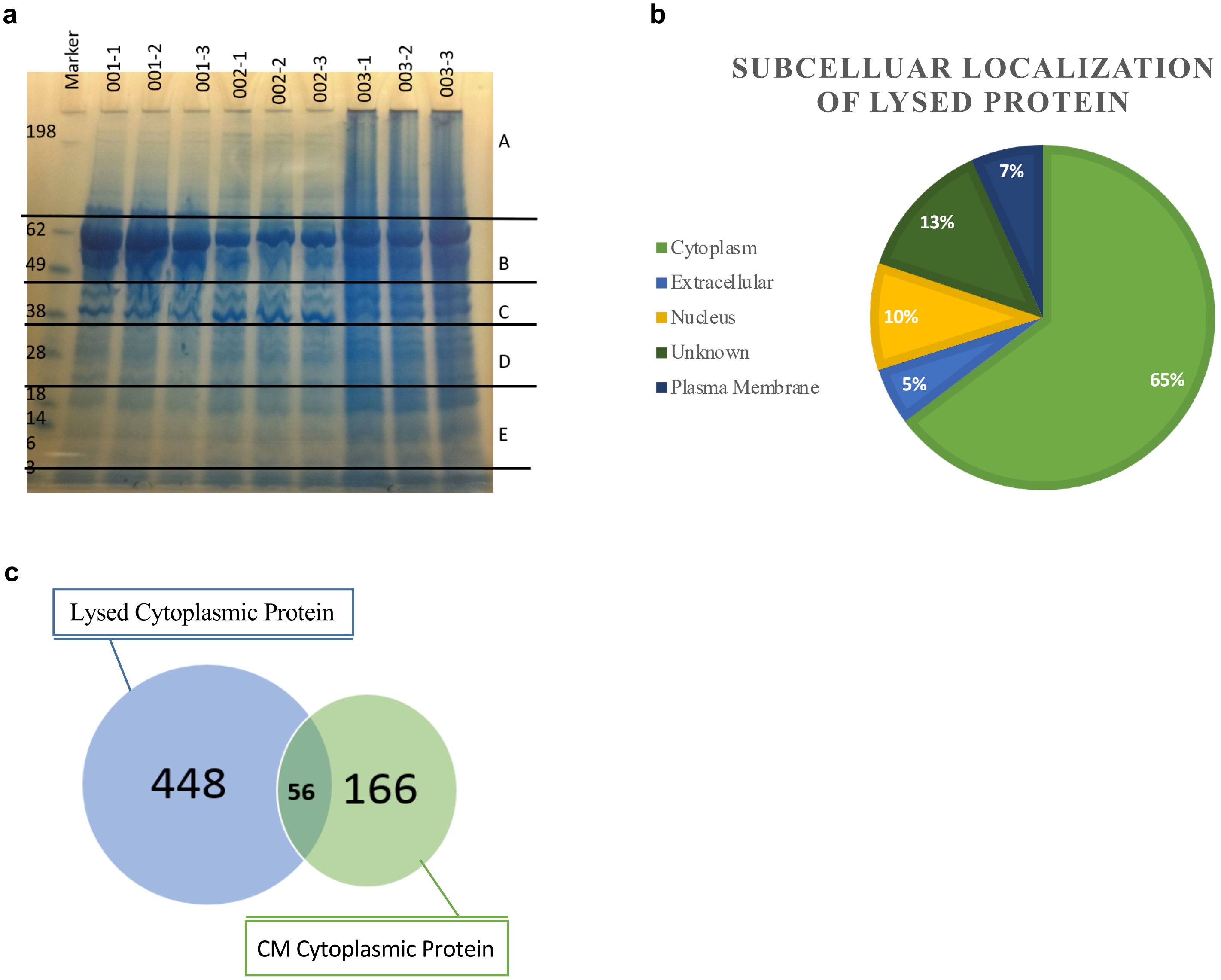

Supplementary Figure 3. Proteomic analysis of lysed LSCs. (**a**) SDS-PAGE gel of LSC lysate. 001= donor 1, 002= donor2, 003= donor 3. (**b**) Quantification of subcellular localization of lysed LSC proteins. (**c**) Comparison of cytoplasmic proteins identified in LSC-Sec versus LSC lysate.

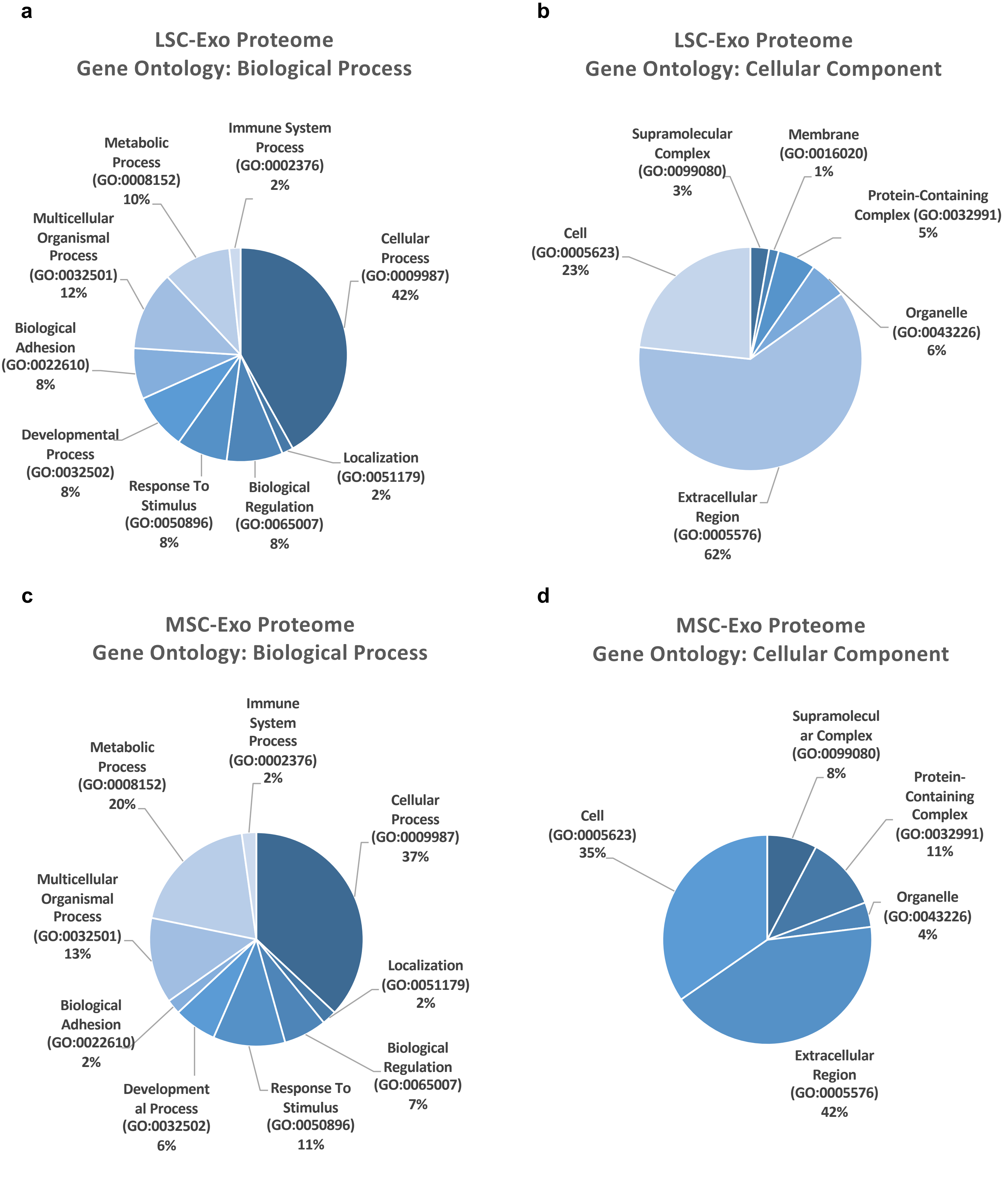

Supplementary Figure 4. Proteomic analysis of LSC-Exosomes and MSC-Exosomes. (**a**) Gene ontology pie chart of biological process associated with LSC-Exo. (**b**) Gene ontology pie chart of cellular process associated with LSC-Exo. (**c**) Gene ontology pie chart of biological process associated with MSC-Exo. (**d**) Gene ontology pie chart of cellular process associated with MSC-Exo.

a

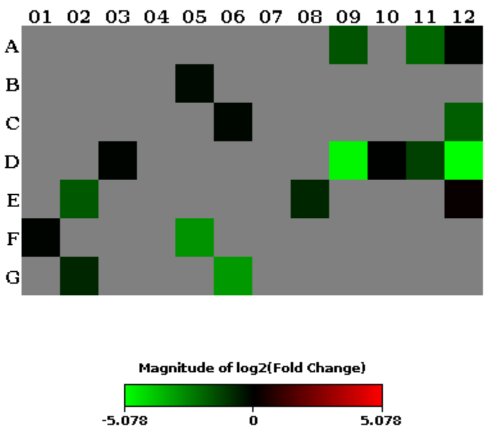

Layout	01	02	03	04	05	06	07	08	09	10	11	12
A	hsa-miR-142-5p **1.23** C	hsa-miR-9-5p **1.23** C	hsa-miR-150-5p **1.23** C	hsa-miR-27b-3p **1.23** C	hsa-miR-101-3p **1.23** C	hsa-let-7d-5p **1.23** C	hsa-miR-103a-3p **1.23** C	hsa-miR-16-5p **1.23** C	hsa-miR-26a-5p **-3.20** B	hsa-miR-32-5p **1.23** C	hsa-miR-26b-5p **-4.08** B	hsa-let-7g-5p **-1.06** B
B	hsa-miR-30c-5p **1.23** C	hsa-miR-96-5p **1.23** C	hsa-miR-185-5p **1.23** C	hsa-miR-142-3p **1.23** C	hsa-miR-24-3p **-1.14** B	hsa-miR-155-5p **1.23** C	hsa-miR-146a-5p **1.23** C	hsa-miR-425-5p **1.23** C	hsa-miR-181b-5p **1.23** C	hsa-miR-302b-3p **1.23** C	hsa-miR-30b-5p **1.23** C	hsa-miR-21-5p **1.23** C
C	hsa-miR-30e-5p **1.23** C	hsa-miR-200c-3p **1.23** C	hsa-miR-15b-5p **1.23** C	hsa-miR-223-3p **1.23** C	hsa-miR-194-5p **1.23** C	hsa-miR-210-3p **-1.09** B	hsa-miR-15a-5p **1.23** C	hsa-miR-181a-5p **1.23** C	hsa-miR-125b-5p **1.23** C	hsa-miR-99a-5p **1.23** C	hsa-miR-28-5p **1.23** C	hsa-miR-320a **-3.70** B
D	hsa-miR-125a-5p **1.23** C	hsa-miR-29b-3p **1.23** C	hsa-miR-29a-3p **-1.07** B	hsa-miR-141-3p **1.23** C	hsa-miR-19a-3p **1.23** C	hsa-miR-18a-5p **1.23** C	hsa-miR-374a-5p **1.23** C	hsa-miR-423-5p **1.23** C	hsa-let-7a-5p **-30.65** A	hsa-miR-124-3p **-1.03** B	hsa-miR-92a-3p **-2.41** B	hsa-miR-23a-3p **-33.77** A
E	hsa-miR-25-3p **1.23** C	hsa-let-7e-5p **-3.34** B	hsa-miR-376c-3p **1.23** C	hsa-miR-126-3p **1.23** C	hsa-miR-144-3p **1.23** C	hsa-miR-424-5p **1.23** C	hsa-miR-30a-5p **1.23** C	hsa-miR-23b-3p **-1.67** B	hsa-miR-151a-5p **1.23** C	hsa-miR-195-5p **1.23** C	hsa-miR-143-3p **1.23** C	hsa-miR-30d-5p **1.11** B
F	hsa-miR-191-5p **-1.06** B	hsa-let-7i-5p **1.23** C	hsa-miR-302a-3p **1.23** C	hsa-miR-222-3p **1.23** C	hsa-let-7b-5p **-7.66** B	hsa-miR-19b-3p **1.23** C	hsa-miR-17-5p **1.23** C	hsa-miR-93-5p **1.23** C	hsa-miR-186-5p **1.23** C	hsa-miR-196b-5p **1.23** C	hsa-miR-27a-3p **1.23** C	hsa-miR-22-3p **1.23** C
G	hsa-miR-130a-3p **1.23** C	hsa-let-7c-5p **-1.68** B	hsa-miR-29c-3p **1.23** C	hsa-miR-140-3p **1.23** C	hsa-miR-128-3p **1.23** C	hsa-let-7f-5p **-8.27** B	hsa-miR-122-5p **1.23** C	hsa-miR-20a-5p **1.23** C	hsa-miR-106b-5p **1.23** C	hsa-miR-7-5p **1.23** C	hsa-miR-100-5p **1.23** C	hsa-miR-302c-3p **1.23** C
H	cel-miR-39-3p **1.23** C	cel-miR-39-3p **1.23** C	SNORD61 **1.23** C	SNORD68 **1.23** C	SNORD72 **1.23** C	SNORD95 **1.23** C	SNORD96A **1.23** C	RNU6-6P **1.23** C	miRTC **1.23** C	miRTC **1.23** C	PPC **1.76**	PPC **1.63**

b

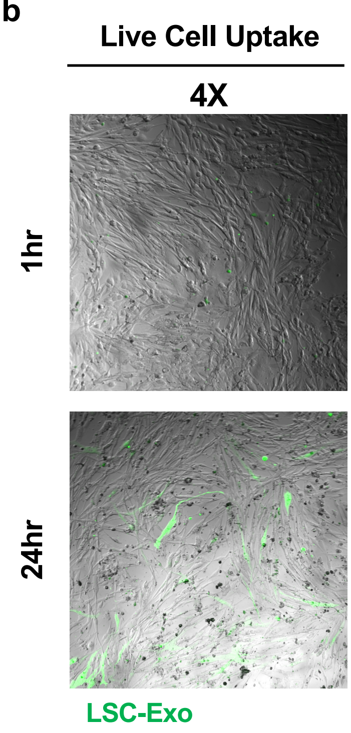

c

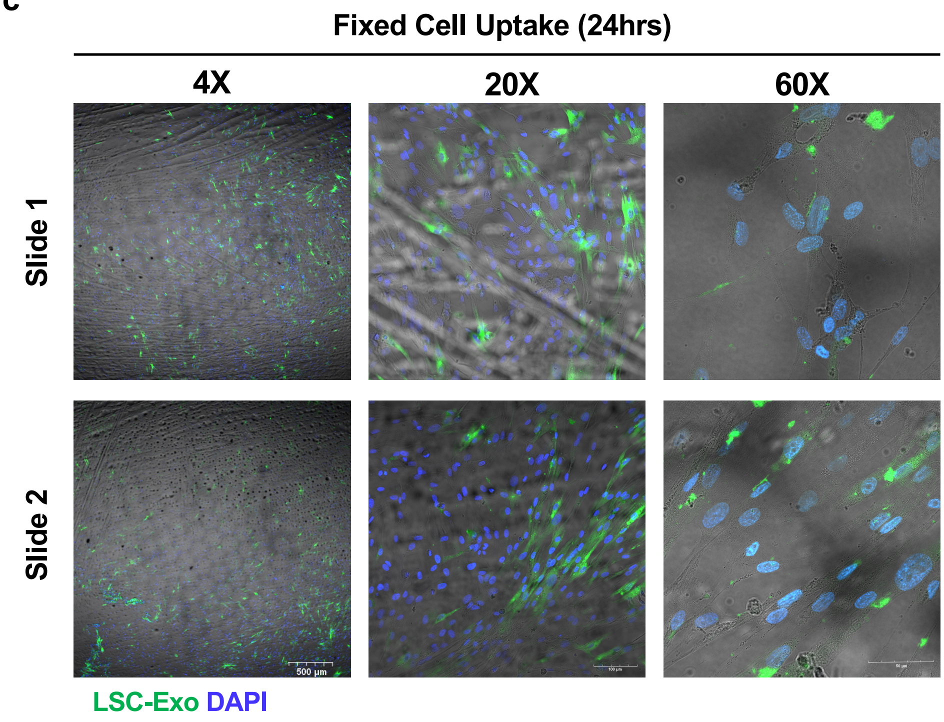

Supplementary Figure 5. LSC-Exosome analysis for cryostability and cellular uptake. (**a**) Heatmap of miRNA microarray analysis of fresh verse frozen LSC-Exo with corresponding plate layout. (**b**) Live cell uptake of DiO labeled LSC-Exo at 1hr and 24hr. (**c**) DiO labeled LSC-Exo with DAPI stained cell nuclei. 4X scale bar= 500µm; 20X scale bar= 100µm; 60X scale bar= 50µm.

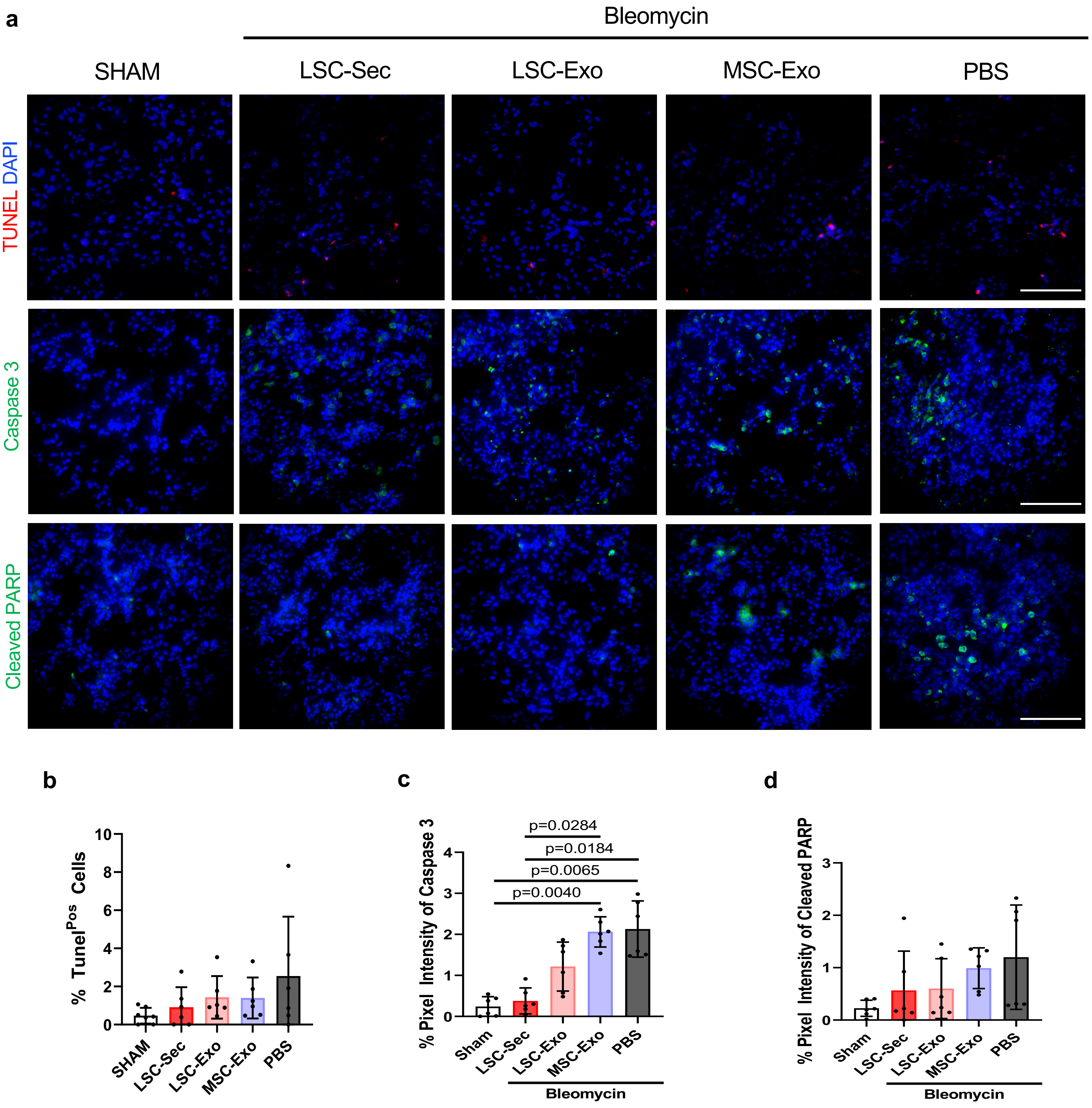

Supplementary Figure 6. LSC-Exo study immunostained analysis of apoptotic cells. (**a**) Representative Tunel (top), caspase 3 (middle), and cleaved PARP (bottom) immunostained analysis of apoptotic cells for each treatment group (**b-d**) and quantification of percent of Tunel positive cells (**b**), percent pixel intensity of Caspase 3 (**c**), and percent pixel intensity of cleaved PARP (**d**); Scale bar= 100µm; each dot represents data from one animal; n=6 biological independent animals. Throughout, data are mean ± s.d. P-value as indicated by non-parametric one-way ANOVA. Scale bars= 100µm

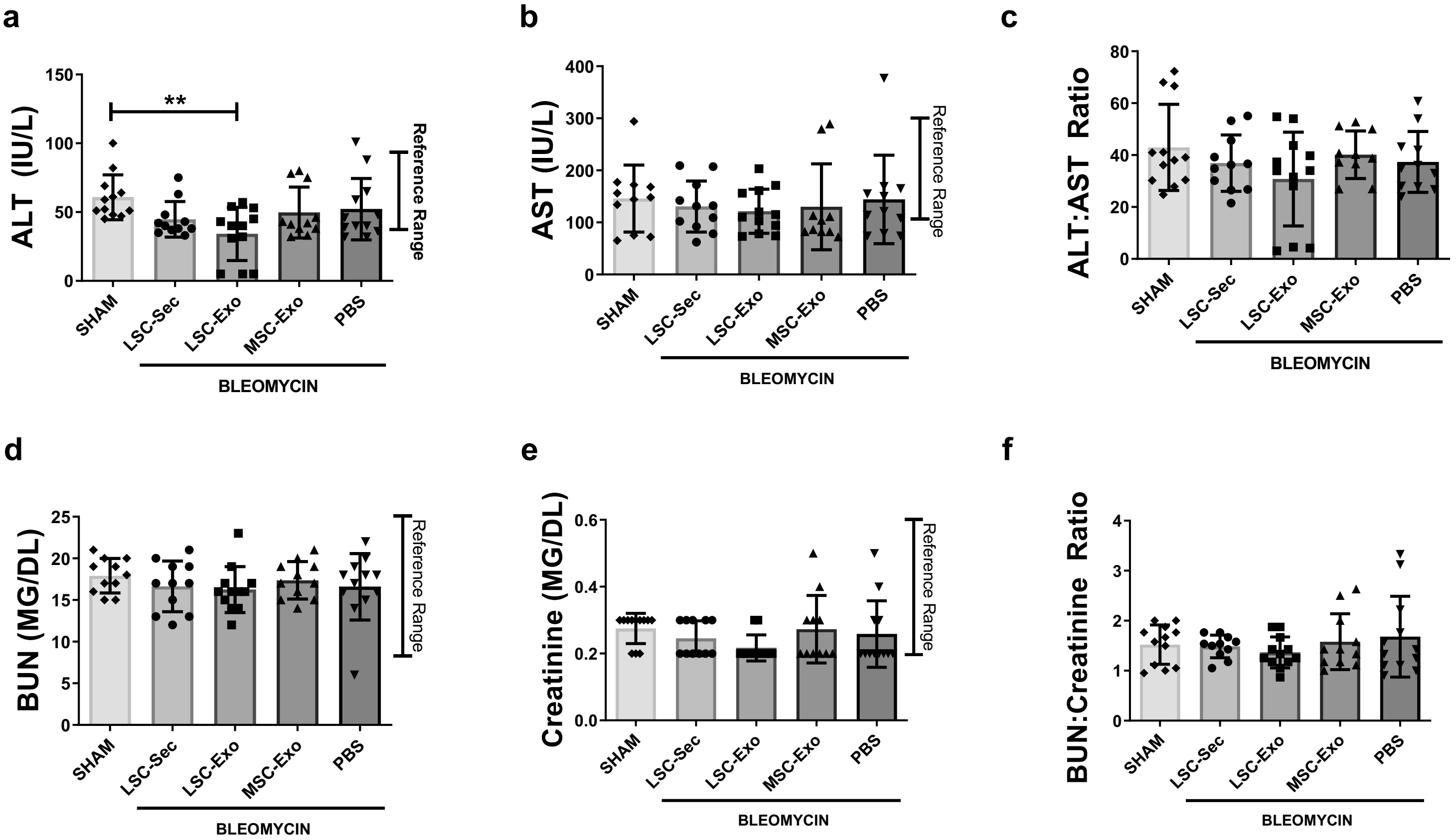

Supplementary Figure 7. Blood biochemistry analysis. (**a-c**) liver enzymes alanine transaminase (ALT) (**a**), aspartate transaminase (AST) (**b**), ratio of ALT: AST (**c**) and (**d-f**) kidney metabolite blood urea nitrogen (BUN) (**d**), creatinine (**e**) and the ratio of BUN: creatinine (**f**); each data point represents data from one animal; n=11 biological independent animals. Throughout, data are mean ± s.d. P-value as indicated by non-parametric one-way ANOVA.
**P≤0.01.

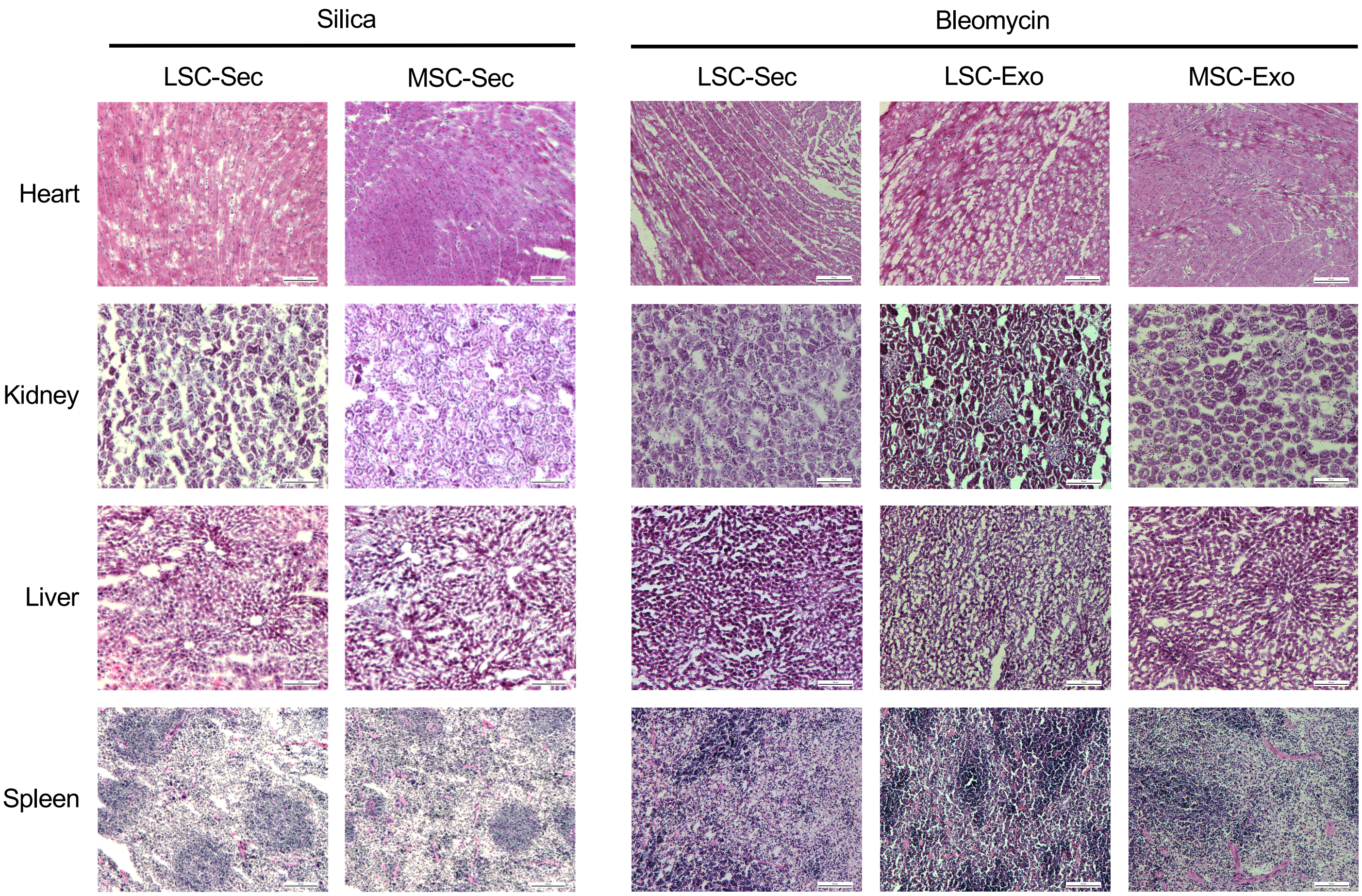

Supplementary Figure 8. Representative H&E staining of heart, kidney, liver and spleen in secretome and exosome treated animals in both the A/J mice silica study and SD rats bleomycin study showing no tumors.

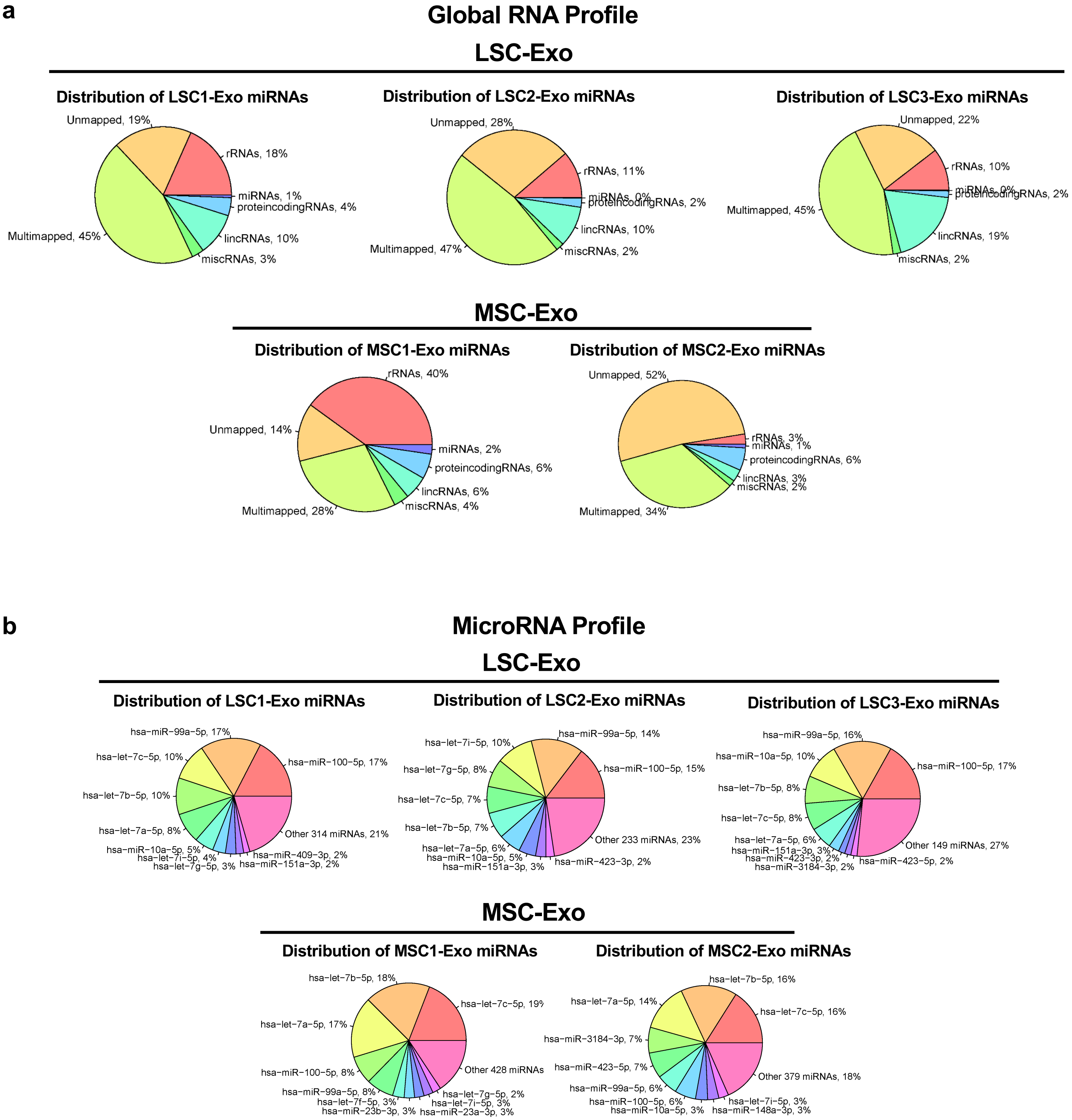

Supplementary Figure 9. RNA sequencing analysis of LSC-Exo and MSC-Exo samples. (**a**) Global RNA profile of LSC-Exo from the three donor cell lines and MSC-Exo from two different cell lines. (**b**) Top 10 miRNAs in each of the exosome samples.

Cell Line	Sex	Age	Race	Smoker	Lung Disease	Cause of Death
Whole Lung Donor 1	Female	50	Hispanic	No	No	Anoxia; 2nd Cardiovascular
Whole Lung Donor 2	Female	52	Black	No	No	Cerebrovascular Accident
Whole Lung Donor 3	Male	18	Hispanic	No	No	Head Trauma; 2nd Self-Inflicted Gunshot Wound

Supplementary Table 1: Cell Line Donor Information
