# Supplementary material for: Inhalation of lung spheroid cell secretome and exosomes promotes lung repair in pulmonary fibrosis
Source: Nat Commun. 2020 Feb 28;11:1064. doi: 10.1038/s41467-020-14344-7 (PMC7048814; doi:10.1038/s41467-020-14344-7)
Supplement: Supplementary file 2 — Description of Additional Supplementary Files [file 41467_2020_14344_MOESM2_ESM.docx]

**Description of Supplementary Files**

**File Name:** Supplementary Data 1

**Description:** Extracellular Protein Identified in All Three Donor LSC-Secretome

**File Name:** Supplementary Data 2

**Description:** LSC Proteome Homology

**File Name:** Supplementary Data 3

**Description:** Proteins Identified in LSC-Exosomes

**File Name:** Supplementary Data 4

**Description:** Proteins Identified in MSC-Exosomes

**File Name:** Supplementary Data 5

**Description:** Adverse Events

**File Name:** Supplementary Data 6

**Description:** Top 25 miRNA Profile in LSC-Exo and MSC-Exo

**File Name:** Supplementary Data 7

**Description:** miRNA Differentially Expression Table

**File Name:** Supplementary Data 8

**Description:** miRNA Sequence Homology
