# Supplementary material for: Inhalation of lung spheroid cell secretome and exosomes promotes lung repair in pulmonary fibrosis
Source: Nat Commun. 2020 Feb 28;11:1064. doi: 10.1038/s41467-020-14344-7 (PMC7048814; doi:10.1038/s41467-020-14344-7)
Supplement: Supplementary file 11 — Reporting Summary [file 41467_2020_14344_MOESM11_ESM.pdf]

nature research

Corresponding author(s): Ke Cheng
Last updated by author(s): Nov 14, 2019

Reporting Summary

Nature Research wishes to improve the reproducibility of the work that we publish. This form provides structure for consistency and transparency in reporting. For further information on Nature Research policies, see Authors & Referees and the Editorial Policy Checklist .

Statistics

For all statistical analyses, confirm that the following items are present in the figure legend, table legend, main text, or Methods section.

n/a	Confirmed	
☐	☒	The exact sample size (*n*) for each experimental group/condition, given as a discrete number and unit of measurement
☐	☒	A statement on whether measurements were taken from distinct samples or whether the same sample was measured repeatedly
☐	☒	The statistical test(s) used AND whether they are one- or two-sided<br>*Only common tests should be described solely by name; describe more complex techniques in the Methods section.*
☒	☐	A description of all covariates tested
☐	☒	A description of any assumptions or corrections, such as tests of normality and adjustment for multiple comparisons
☐	☒	A full description of the statistical parameters including central tendency (e.g. means) or other basic estimates (e.g. regression coefficient) AND variation (e.g. standard deviation) or associated estimates of uncertainty (e.g. confidence intervals)
☐	☒	For null hypothesis testing, the test statistic (e.g. *F*, *t*, *r*) with confidence intervals, effect sizes, degrees of freedom and *P* value noted<br>*Give P values as exact values whenever suitable.*
☒	☐	For Bayesian analysis, information on the choice of priors and Markov chain Monte Carlo settings
☒	☐	For hierarchical and complex designs, identification of the appropriate level for tests and full reporting of outcomes
☒	☐	Estimates of effect sizes (e.g. Cohen's *d*, Pearson's *r*), indicating how they were calculated

Our web collection on statistics for biologists contains articles on many of the points above.

Software and code

Policy information about availability of computer code

Data collection	Exosome particle analysis was collected on the Nanoparticle Tracking Analysis (NTA; NanoSight).<br>Electron microscopy images was collected on the JEOL JEM-2000FX.<br>Fluorescent imaging was collected on the Olympus IX81.<br>Western blot images was collected on the BioRad ChemiDoc Imaging System.<br>Pulmonary function test was collected on the FlexiVent FX Module 4 version 7.6.<br>Mass spectrometry data was collected on the LTQ Orbitrap Elite and the Q Exactive HF-X Hybrid Quadrupole-Orbitrap.<br>RNA sequence data was collected on the Illumina NextSeq500.<br>miRNA PCR array was collected on the Roche LightCycler480.<br>Blood biochemistry data was collected on the Cobas C501 by Roche.
Data analysis	Statistical analysis was performed using GraphPad Prism version 8.0.<br>Proteomic data was analyzed using Proteome Discover version 1.4 for processing raw data files, Mascot version 1.4.1.14 for comparison against the Homo sapiens UniProt database, Scaffold version 4.8.4 for quantification of peptides/proteins and statistical analysis, Panther (Protein Analysis Through Evolutionary Relationships), and DAVID (The Database for Annotation, Visualization and Integrated Discovery) for functional and pathway analysis.<br>Genomic data was analyzed using Illumina bcl2fastq for processing raw data, mirDeep2, and Bowtie for read cluster and alignment to human rRNAs. EdgeR and limma R packages was used for differential expression analysis.

For manuscripts utilizing custom algorithms or software that are central to the research but not yet described in published literature, software must be made available to editors/reviewers. We strongly encourage code deposition in a community repository (e.g. GitHub). See the Nature Research guidelines for submitting code & software for further information.

Data

Policy information about availability of data

All manuscripts must include a data availability statement. This statement should provide the following information, where applicable:

- Accession codes, unique identifiers, or web links for publicly available datasets
- A list of figures that have associated raw data
- A description of any restrictions on data availability

Genomic data has been submitted to GEO with accession ID: GSE136263. RNAseq data has not been deposited to any online databases, but can be made available upon request.

Field-specific reporting

Please select the one below that is the best fit for your research. If you are not sure, read the appropriate sections before making your selection.

[x] Life sciences [ ] Behavioural & social sciences [ ] Ecological, evolutionary & environmental sciences

For a reference copy of the document with all sections, see nature.com/documents/nr-reporting-summary-flat.pdf

Life sciences study design

All studies must disclose on these points even when the disclosure is negative.

Sample size	All experiments were performed at least in triplicate independent measurements or more as indicated in the text.<br>Sample size for in-vivo experiment was determined by power analysis using statistics from previous experiments and preliminary experiments.<br>Bleomycin CD1 LSC-Sec Study n= 12-16 per group<br>Silica A/J LSC-Sec Study n= 10 per group<br>Bleomcyin SD LSC-Exo Study n= 11-12 per group
Data exclusions	Pulmonary function data was excluded for analysis from animals that did not have all three baseline, midpoint, and endpoint measurement.
Replication	All experiments in this study was independently replicated, with a minimum of three biological and technical replicates as indicated in the text or corresponding figure legends.
Randomization	All animals were randomly allocated into each experimental groups for all three animal study.
Blinding	Investigators were not blinded to allocation during experiments and outcomes assessment. However, Ashcroft score was performed by averaging the scores from one blinded scorer and one non-blinded scorer. Blood biochemistry analysis was performed by a blinded third-party.

Reporting for specific materials, systems and methods

We require information from authors about some types of materials, experimental systems and methods used in many studies. Here, indicate whether each material, system or method listed is relevant to your study. If you are not sure if a list item applies to your research, read the appropriate section before selecting a response.

Materials & experimental systems

n/a	Involved in the study	
[ ]	[x]	Antibodies
[ ]	[x]	Eukaryotic cell lines
[x]	[ ]	Palaeontology
[ ]	[x]	Animals and other organisms
[x]	[ ]	Human research participants
[x]	[ ]	Clinical data

Methods

n/a	Involved in the study	
[x]	[ ]	ChIP-seq
[x]	[ ]	Flow cytometry
[x]	[ ]	MRI-based neuroimaging

Antibodies

Antibodies used	Immunoblotting was performed with the follow antibody: CD63 (NB100-77913, Novus), CD81 (SAB3500454, Sigma-Aldrich), TSG101(MA1-23296, Thermo Fisher), MMP2 (ab86607, Abcam), αSMA (ab5694, Abcam), SMAD3 (MA5-14939, ThermoFisher Scientific), CD63 (nb100-77913, Novus Biologicals), CD81 (MA5-13548, ThermoFisher Scientific), GAPDH (MA5-15738-HRP, ThermoFisher Scientific), beta-actin (MA5-15739, ThermoFisher Scientific), CD9 (MA5-31980, ThermoFisher Scientific), AQP5 (ab78486, Abcam), ProSPC (ab40879, Abcam), and vWF (ab96340, Abcam).

	Immunostaining was performed with the following antibodies: AQP5 (ab78486, Abcam), ProSPC (ab90716, Abcam), vWF (F3520, Sigma-Aldrich), αSMA (ab5694, Abcam), PARP (44-698G, ThermoFisher Scientific), Caspase 3 (ab13847, Abcam), vimentin (ab20346, Abcam), CD105 (ab44967, Abcam), EpCAM (324204, Biolegend), and Uteroglobin (ab140663, Abcam).<br><br>Tunel staining was performed using the In-Situ Cell Death Detection Kit (Roche Diagnostics, Mannheim, Germany) according to the manufacturer's instructions.
Validation	All antibodies used are commercially available and have been validated for the application used by the manufacturer. Specific references for each antibody can be found on the manufactures website. Appropriate controls were used for all immunoblotting and immunostaining.

Eukaryotic cell lines

Policy information about cell lines

Cell line source(s)	All human Lung Spheroid Cells (LSCs) were generated from healthy whole lung donors acquired from the Cystic Fibrosis and Pulmonary Disease Research and Treatment Center at the University of North Carolina at Chapel Hill and expanded as described in Henry, Stem Cells Transl. Med. (2015), Dinh, Respir. Res. 2017), and Cores, Stem Cells Transl. Med. (2017). Human bone marrow-derived Mesenchymal Stem Cells (MSCs) were purchased from the American Type Culture Collection (ATCC).
Authentication	All cell lines were analyzed by flow cytometry for the appropriate markers before use. Reference Dinh, Respir. Res. 2017.
Mycoplasma contamination	All cell lines tested negative for mycoplasma.
Commonly misidentified lines (See ICLAC register)	None of the cell lines used are listed in the ICLAC database of commonly misidentified cell lines.

Animals and other organisms

Policy information about studies involving animals; ARRIVE guidelines recommended for reporting animal research

Laboratory animals	Six to eight week old male CD1 mice [Crl: CD1(ICR)] were obtained from Charles River Laboratory (Massachusetts, USA).<br>Six to eight week old male and female CD (SD) IGS rats [Crl: CD(SD)] were obtained from Charles River Laboratory (Massachusetts, USA).<br>Six to eight week old male A/J mice were obtained from Jackson Laboratory (Maine, USA).
Wild animals	This study did not involve any wild animals.
Field-collected samples	This study did not involve any field-collected samples.
Ethics oversight	All studies complied with the requirements of the Institutional Animal Care and Use Committee at North Carolina State University.

Note that full information on the approval of the study protocol must also be provided in the manuscript.
